# Tenascin-C modulates alveolarization in bronchopulmonary dysplasia

**DOI:** 10.1186/s41232-024-00330-9

**Published:** 2024-03-28

**Authors:** Wei Liu, Yu Mao, Qianru Lv, Keyu Lu, Chunyu Yin, Rui Cheng, Mingshun Zhang

**Affiliations:** 1 Department of Neonatal Medical Center, Children’s Hospital of Nanjing Medical University, Nanjing, China; 2 Department of Immunology, NHC Key Laboratory of Antibody Technique, Jiangsu Key Laboratory of Pathogen Biology, Nanjing Medical University, Nanjing, China

**Keywords:** Bronchopulmonary dysplasia, Extracellular matrix, Tenascin-C, Hyperoxia, Epithelial cells, ICAM-1

## Abstract

Bronchopulmonary dysplasia (BPD) is a chronic lung disease characterized by retarded alveolarization. Tenascin-C (TN-C), an extracellular matrix glycoprotein and soluble molecule, is involved in tissue morphogenesis. In the present study, we demonstrated that the level of TN-C in lung tissues was greater in a mouse model of BPD induced by 85% oxygen. TN-C deficiency, however, impaired alveolarization in the hyperoxia-induced BPD model. In contrast, a functional TN-C blocking antibody ameliorated alveolar dysplasia in BPD-like mice. Mechanistically, hyperoxia increased the soluble TN-C (sTN-C) released from respiratory epithelial cells. On one hand, low-dose sTN-C promoted lung epithelial cell proliferation and migration, which was mediated by ICAM-1. On the other hand, high-dose sTN-C hindered the proliferation and migration of epithelial cells. Overall, this study revealed that TN-C plays a dual role in lung alveolarization and that TN-C may be a target in BPD therapy.

**Supplementary Information:**

The online version contains supplementary material available at 10.1186/s41232-024-00330-9.

## Introduction

Bronchopulmonary dysplasia (BPD) is a syndrome in which late lung development is retarded and associated with impeded alveolarization and impaired pulmonary vascularization [1, 2]. Due to different risk factors, including infection, hyperoxia and mechanical ventilation, BPD is an important cause of morbidity and mortality in preterm infants [3, 4]. The histopathology of "new" BPD is characterized by large, simplified alveolar structures, variable stromal cells, and/or fibroplasia [5]. Alveolar maturation largely depends on the behavior of alveolar epithelial cells, including cell proliferation and migration [6]. An appropriate and well-structured extracellular matrix (ECM) can provide a scaffold to guide alveolar maturation [7–9]. However, extensive remodeling of the ECM can result in alveolar hypoplasia and therefore contributes to the pathogenesis of BPD [9–11].

Tenascin-C (TN-C) is a large hexameric ECM glycoprotein produced by both epithelial and mesenchymal cells that has paracrine and autocrine regulatory functions on cell migration, proliferation, and differentiation [12–14]. TN-C is transiently expressed during embryonic development and certain pathological conditions, such as inflammation and tumorigenesis, whereas virtually no TN-C expression is observed in normal, fully developed organs [15, 16]. TN-C expression has two peaks during mammalian lung development, with the first peak occurring during airway branching and the second peak occurring in early alveolarization [17]. In the immature fetal lung, TN-C is required for branching morphogenesis [18]. During postnatal lung development, TN-C was noticeably concentrated at the tips of the growing alveolar septa during early alveolation [19]. Moreover, respiratory epithelial cells have been reported to secrete more extracellular soluble TN-C (sTN-C) in an in vitro model of bronchospasm [20], and the sTN-C is increased in numerous pulmonary injury disorders [21–24]. However, the specific roles of TN-C and sTN-C in the proliferation and migration of alveolar epithelial cells and the maturation of alveoli in the development of BPD are largely elusive.

In the present study, we aimed to investigate the role of the ECM protein TN-C, especially sTN-C, in postnatal alveolarization and BPD pathogenesis. Our data demonstrated that TN-C is a double-edged sword in alveolar maturation. HIF-1α-stimulated sTN-C mediates alveolar epithelial cell proliferation and migration, and deficiency of TN-C arrests lung development. However, excessive TN-C is detrimental to lung alveolarization and contributes to the development of BPD, suggesting that a delicate balance in the production of TN-C is vital for lung development.

## Results

### Dynamic changes in ECM proteins during late murine lung development

**Fig. 1:**
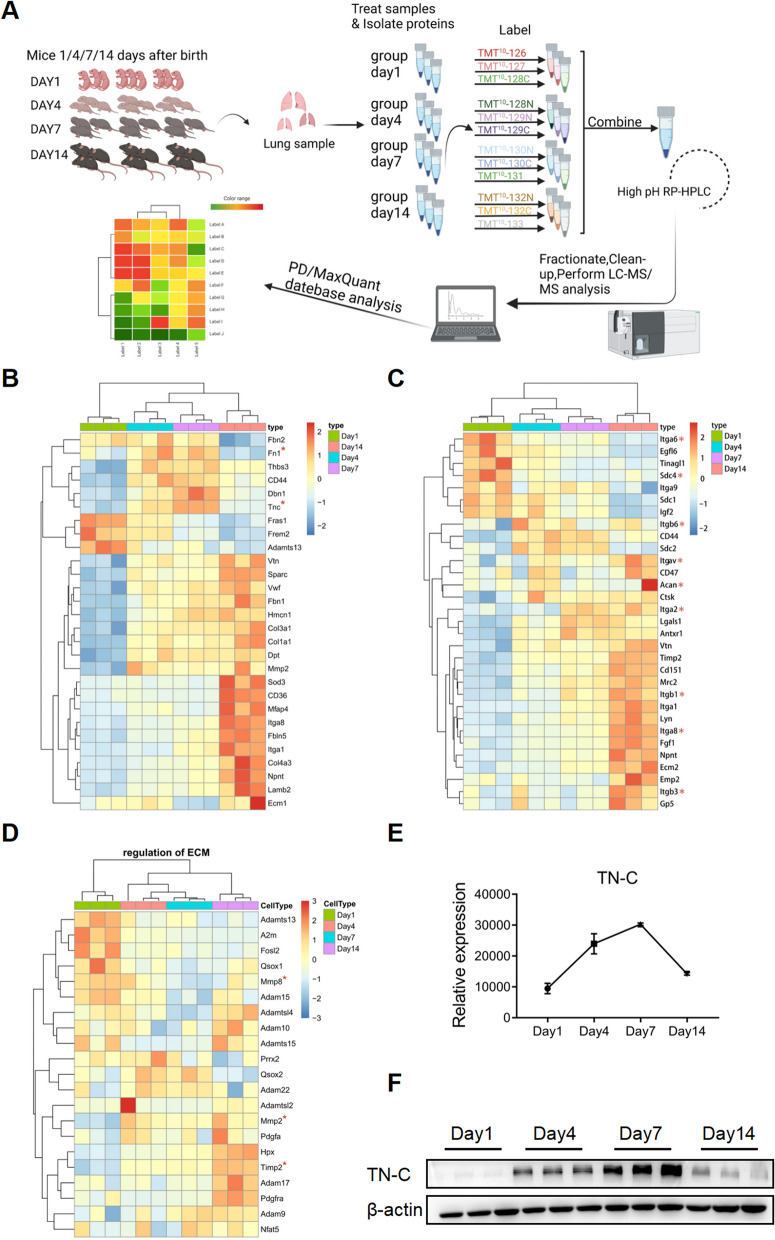
At the early stage of mouse lung development, dynamic changes in the protein expression of ECM components were observed. **A** The workflow of a systems approach to TMT proteomics analysis. **B** Hierarchical clustering and heatmap of differentially expressed ECM proteins in postnatal mice. **C** Heatmaps of ECM protein-associated ligands and TN-C ligands are marked with asterisks. **D** Heatmaps of the regulatory components of ECM proteins. **E** The relative expression of the TN-C protein in the lungs of mice on postnatal days (P) 1, 4, 7 and 14. **F** The expression of the TN-C protein was verified via Western blotting

To investigate alterations in ECM components during the alveolar differentiation stage in the developing lung, we performed proteomic analysis of 36 mice at postnatal days (P) 1, 4, 7, and 14 (Fig. 1A) and identified a total of 7660 proteins. Then, we used bioinformatics methods to screen the top 20 ECM components with the highest expression, including ECM proteins, ECM-related ligand proteins and ECM component regulatory proteins (Fig. 1B-D). We discovered that highly expressed ECM proteins in the early stages of lung development exhibited three expression patterns: (1) a continuous increase, (2) a consistent decrease and (3) an initial increase and subsequent reduction. The ECM component fibronectin was shown in our previous research to increase initially followed by a decrease throughout the early phases of lung development and to be strongly correlated with the development of BPD [7]. As a result, we focused the proteins on the 3rd pattern, especially the TN-C protein, in the proteomic analysis for subsequent investigation (Fig. 1E). The trend in the expression of the TN-C protein was validated by Western blotting; the level of the TN-C protein first increased gradually (peaking at P7) and then decreased to an undetectable level (P14) (Fig. 1F).

### TN-C expression was aberrant in the lungs of BPD-like mice

**Fig. 2:**
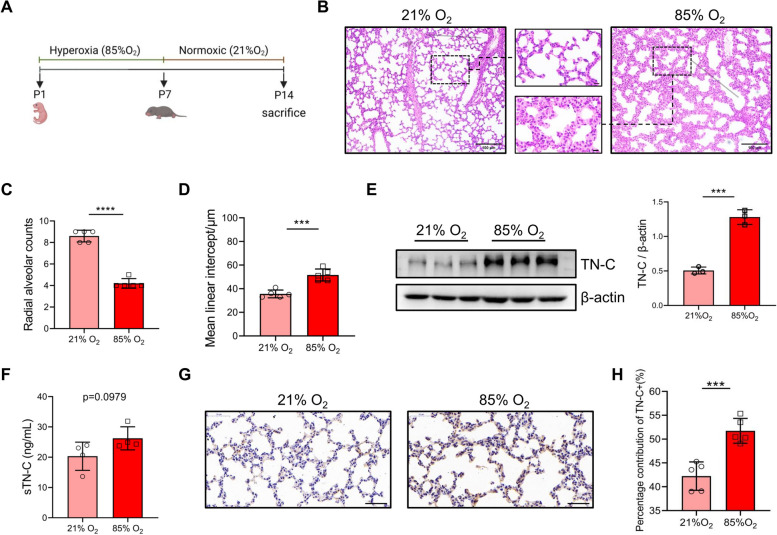
Increased TN-C in the early stage of BPD. **A** To establish the BPD model, newborn mice were exposed to 85% oxygen from postnatal day (P) 1 to P7. Chronic hyperoxic lung injury was evident in this BPD model, and the mice were housed under normoxic conditions. Flow chart showing the BPD model. **B**-**D** Lung morphometry was analyzed via H&E staining of tissue samples from surviving pups at P14. Scale bars = 100 μm. In the enlarged squares, scale bars = 10 μm. **E**–**F** TN-C expression in pulmonary tissues at P14 was measured by ELISA and Western blotting. **G**-**H** Representative sections of immunohistochemical analysis of TN-C from lungs developing in 21% oxygen or 85% oxygen at P14. Five animals/group were evaluated. Scale bars = 50 μm. The data are expressed as the means ± SEMs. *P < 0.05, ** *P* < 0.01, ***P < 0.001, **** *P* < 0.0001

To demonstrate the significance of this specific expression pattern of TN-C protein in BPD, we established a murine model of BPD with postnatal hyperoxia. As depicted in Fig. 2A, pups were exposed to either indoor air (21% oxygen, control group) or 85% oxygen (BPD group) until the 7th day and raised to 14th day in the 21% oxygen for the lung maturation. Compared with those in the control group, histological analysis in the BPD group demonstrated arrested alveolar development with significantly decreased alveolarization regions (Fig. 2B-D). In contrast, the alveolar septa in the hyperoxia group were significantly thickened with decreased secondary septa formation as shown in enlarged squares in Fig. 2B. To explore whether the TN-C protein level changes during the pathogenesis of alveolar epithelial cell injury in BPD, we measured the expression of the TN-C protein in pulmonary tissues using Western blotting and ELISA. The TN-C protein level was significantly greater in the hyperoxia group than in the normoxia group (Fig. 2E-F). Furthermore, as observed in the immunohistochemical sections, the expression of the TN-C protein in the thickened alveolar septum was more pronounced in the hyperoxia-treated group. (Fig. 2G-H). Overall, the abnormal and persistent high expression of the TN-C protein suggested that TN-C may be involved in the early impaired alveolarization stage of BPD.

### Hyperoxia promotes the release of sTN-C from respiratory epithelial cells through HIF-1α

**Fig. 3:**
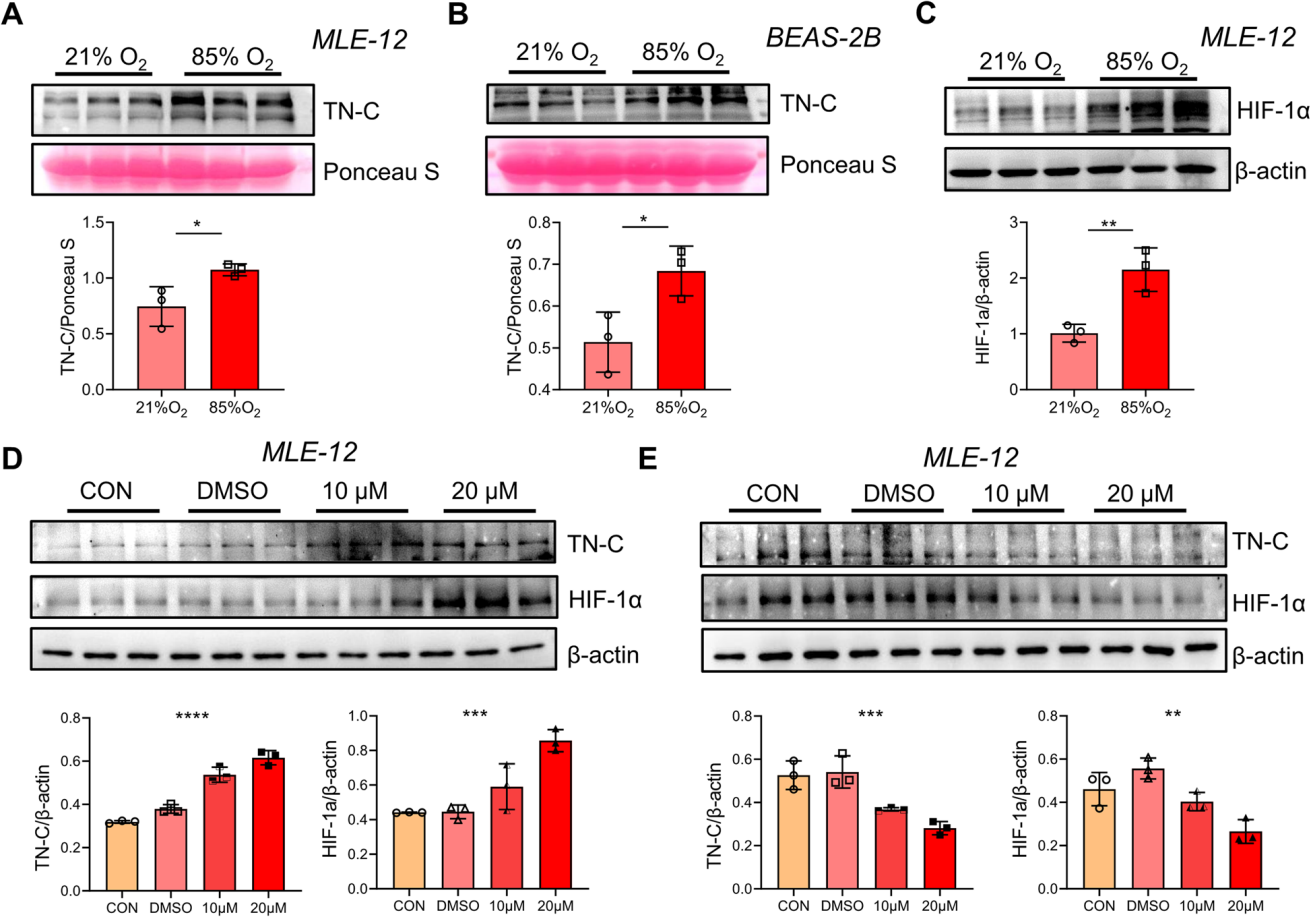
HIF-1α directly induces soluble TN-C expression in epithelial cells. **A**-**B** Soluble TN-C (sTN-C) was measured via Western blotting, and Ponceau S was used as a loading control to assess the total protein concentration in the cell culture supernatant. **C** HIF-1α protein expression in MLE-12 cells stimulated with 85% oxygen for 12 h. **D**-**E** TN-C protein expression in MLE-12 cells stimulated with the HIF-1α agonist DMOG under normoxia (**D**) or the HIF-1α inhibitor 2-MeOE2 under 85% hyperoxia (**E**) for 12 h was measured via Western blotting. The density quantification of TN-C or HIF-1α was expressed as a ratio relative to β-actin. *P < 0.05, ** *P* < 0.01, ***P < 0.001, **** *P* < 0.0001

In line with the previous reports [25, 26], TN-C is widely distributed in bronchiolar and alveolar epithelium (Fig. S1). Since hyperoxia can lead to excessive production of TN-C protein in the lung tissues from BPD-like mice, we directly treated lung epithelial cells with hyperoxia (85% oxygen) and found that both human and mouse respiratory epithelial cells (BEAS-2B and MLE-12 cells) secreted more soluble TN-C after hyperoxia exposure (Fig. 3A-B). Intermittent hyperoxia treatment can cause cells to experience relative hypoxia, thereby activating the HIF-1 pathway [27, 28]. Thus, the expression of HIF-1α in mouse alveolar epithelial cells was analyzed by Western blotting. As shown in Fig. 3C, hyperoxia increased the expression of HIF-1α. Furthermore, activation of HIF-1α expression under normoxia promoted increased expression of TN-C in mouse alveolar epithelial cells, whereas treatment with 2-MeOE2, an inhibitor of HIF-1α, suppressed TN-C expression in hyperoxia-treated epithelial cells (Fig. 3D-E). Overall, these results indicated that hyperoxia may promote the secretion of TN-C in lung epithelial cells through the HIF-1α pathway.

### Low-dose sTN-C promotes epithelial cell proliferation and migration

**Fig. 4:**
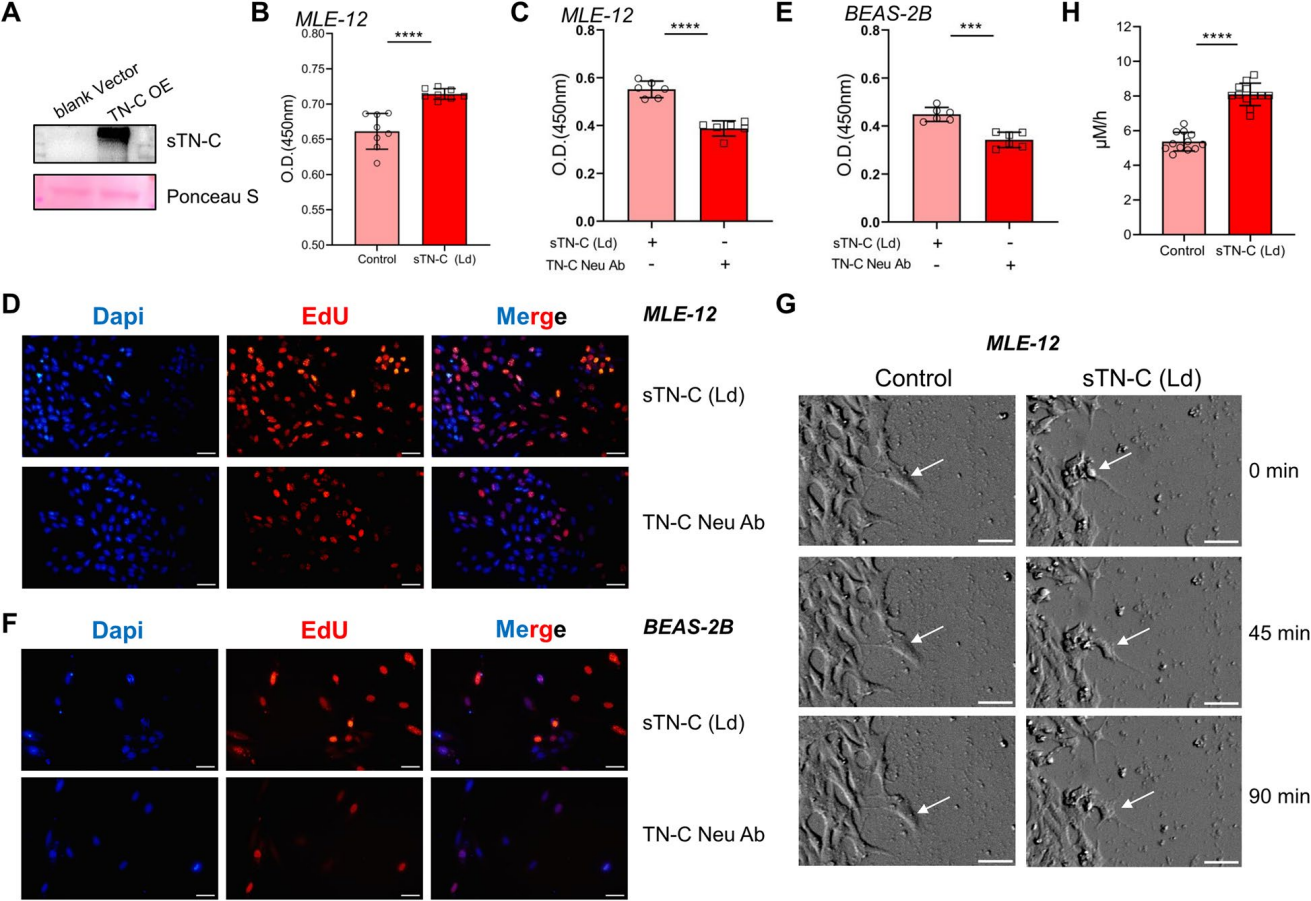
Low-concentration sTN-C supplementation enhanced the proliferation and migration of epithelial cells. **A** The overexpression of sTN-C in the supernatant of transfected 293 T cells was verified by Western blotting. **B** CCK-8 assay for determining the viability of MLE-12 cells incubated for 24 h under normoxia after low-dose sTN-C supplementation (2 ng/mL). **C**, **E**. CCK-8 assay was used to assess the viability of MLE-12 cells or BEAS-2B cells incubated for 24 h under 85% hyperoxia following low-dose supplementation with sTN-C (2 ng/mL) and TN-C neutralizing antibodies. **D**, **F** EdU assays were used to assess the proliferation of MLE-12 and BEAS-2B cells incubated for 24 h under 85% hyperoxia following low-dose supplementation with sTN-C (2 ng/mL) and TN-C neutralizing antibodies (2 ng/mL). Scale bars = 50 μm. **G**-**H** A live cell imaging system was used to observe the migration ability of alveolar epithelial cells at the scratch edge after a 90-min incubation period under normoxia following the addition of low-dose sTN-C (2 ng/mL). Scale bars = 50 μm. The average migration speed of individual cells was analyzed using ImageJ. All experiments were repeated at least 3 times. ***P < 0.001, **** *P* < 0.0001

To investigate the effect of sTN-C on the phenotype of epithelial cells, first, we used 293 T cells to transfect the expression plasmid encoding TN-C or the control plasmid and harvested the transfection supernatant. Western blotting was used to verify the expression of sTN-C (Fig. 4A). We treated MLE-12 cells with sTN-C under normoxic conditions and found that low-dose sTN-C (2 ng/mL) promoted cell proliferation (Fig. 4B). Then, MLE-12 cells were treated with both low-dose sTN-C protein and a TN-C neutralizing antibody under hyperoxia stimulation, and the proliferation of MLE-12 cells was reduced (Fig. 4C-D). This decrease in proliferation was also observed in human airway epithelial cells (Fig. 4E-F). The enhanced migration of cells at the scratch edge after sTN-C supplementation was also clearly observed in serial images taken by live cell imaging (white arrows, Fig. 4 G-H and Supplement Videos). Taken together, these results suggested that low-dose sTN-C promoted lung epithelial cell proliferation and migration.

### *ICAM-1* is required for sTN-C-induced epithelial cell proliferation

**Fig. 5:**
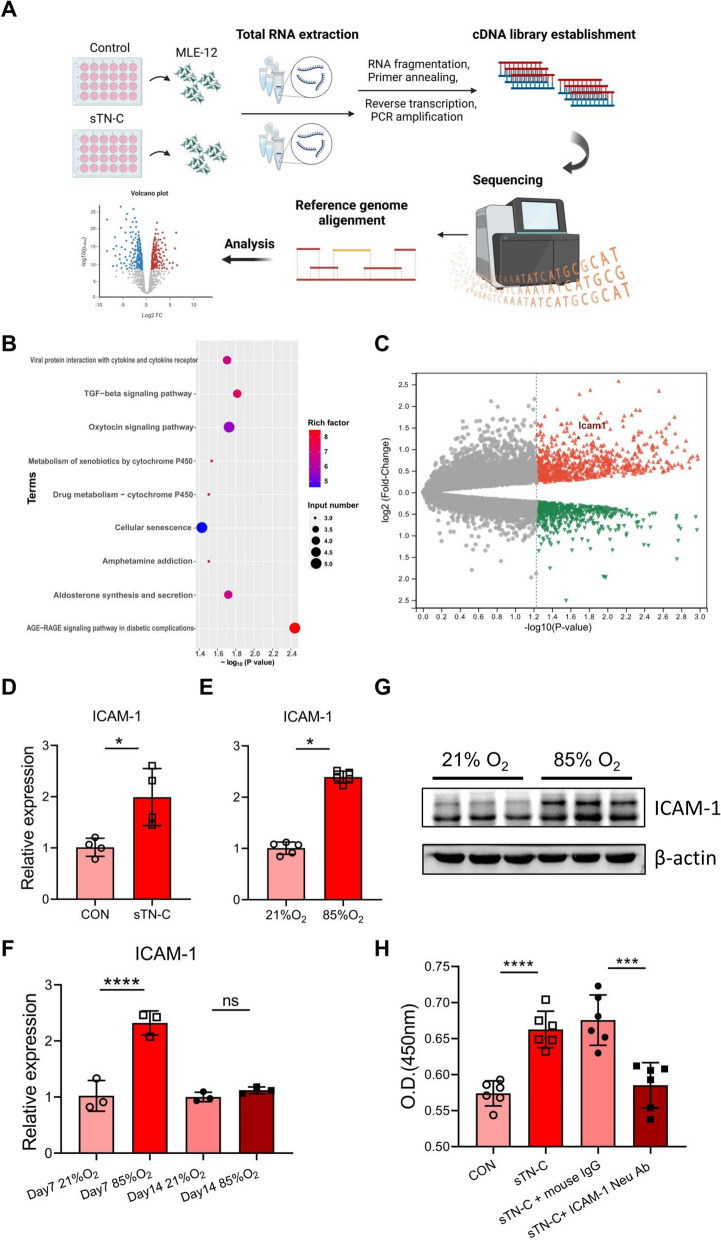
The potential role of ICAM-1 in the proliferation of alveolar epithelial cells stimulated by low-dose sTN-C. 2 ng/mL sTN-C (a blank vector-transfected supernatant as a control) was used to activate MLE-12 cells in 24-well plates for 24 h. Total RNA was extracted using TRIzol, after which RNA sequencing was carried out. **A** Process diagram for transcriptome sequencing. **B** Bubble diagram of KEGG pathway enrichment. The AGE-RAGE signaling pathway was considerably increased in low-dose sTN-C-treated alveolar epithelial cells. **C** Volcano plot showing DEGs in sTN-C-treated alveolar epithelial cells. The upregulated genes are shown in red, and the downregulated genes are shown in green with a fold change > 1.0 and *P* < 0.05. ICAM-1 is marked with a special color. **D** The increase in ICAM-1 RNA expression was verified by qPCR. **E** ICAM-1 RNA expression in MLE-12 cells following 24 h of stimulation with 85% oxygen was measured by qPCR. **F** ICAM-1 RNA expression levels in the lung tissue of BPD model mice at P7 or P14 were measured via qPCR. **G** ICAM-1 protein expression in pulmonary tissues at P14 was measured by Western blotting. **H** In a model of alveolar epithelial cells stimulated with low-dose sTN-C, anti-ICAM-1 monoclonal antibodies (2 μg/mL) and isotype mouse IgG control antibodies (2 μg/mL) were added. The proliferation of MLE-12 cells was assessed using a CCK-8 assay

To explore the mechanisms underlying how sTN-C promotes lung epithelial cell proliferation, total RNA was extracted from MLE-12 cells after low-dose sTN-C stimulation, and transcriptomic analysis was performed, as shown in Fig. 5A. KEGG enrichment analysis was carried out to determine the key pathways associated with the DEGs, and the top 9 enriched signaling pathways are shown in Fig. 5B. Among the pathways, the AGE-RAGE signaling pathway exhibited the most significant enrichment. The AGE-RAGE signaling pathway is involved in early alveolarization and extracellular matrix remodeling and is associated with hyperoxic lung injury after birth [29–31]. The main differentially expressed gene involved in the AGE-RAGE signaling pathway was intercellular adhesion molecule-1 (*ICAM-1*), as illustrated by the volcano plot in Fig. 5C. The receptor for advanced glycation end products (RAGE) acts in concert with *ICAM-1* to mediate pathological processes [32, 33]. Next, we verified that the *ICAM-1* mRNA level in MLE-12 cells indeed increased after stimulation with low-dose sTN-C (Fig. 5D) and that the *ICAM-1* mRNA level was also significantly greater in both hyperoxia-treated MLE-12 cells and the lungs of BPD-like mice (Fig. 5E-F). This increase in the protein level of *ICAM-1* was also observed in the lungs of BPD-like mice (Fig. 5G). To further explore the interaction between sTN-C and ICAM-1, a neutralizing antibody against ICAM-1 was added to the alveolar epithelial cell model before stimulation with low-dose sTN-C. As shown in Fig. 5H, with the inhibition of ICAM-1, the proliferative effect of sTN-C on the epithelium was also rescued. Taken together, our data suggested that ICAM-1 was functionally essential for the promotion of alveolar epithelial cell proliferation by sTN-C and may play a synergistic role with sTN-C in the early stage of BPD.

### TN-C deficiency exacerbates hyperoxia-induced inhibition of lung development

**Fig. 6:**
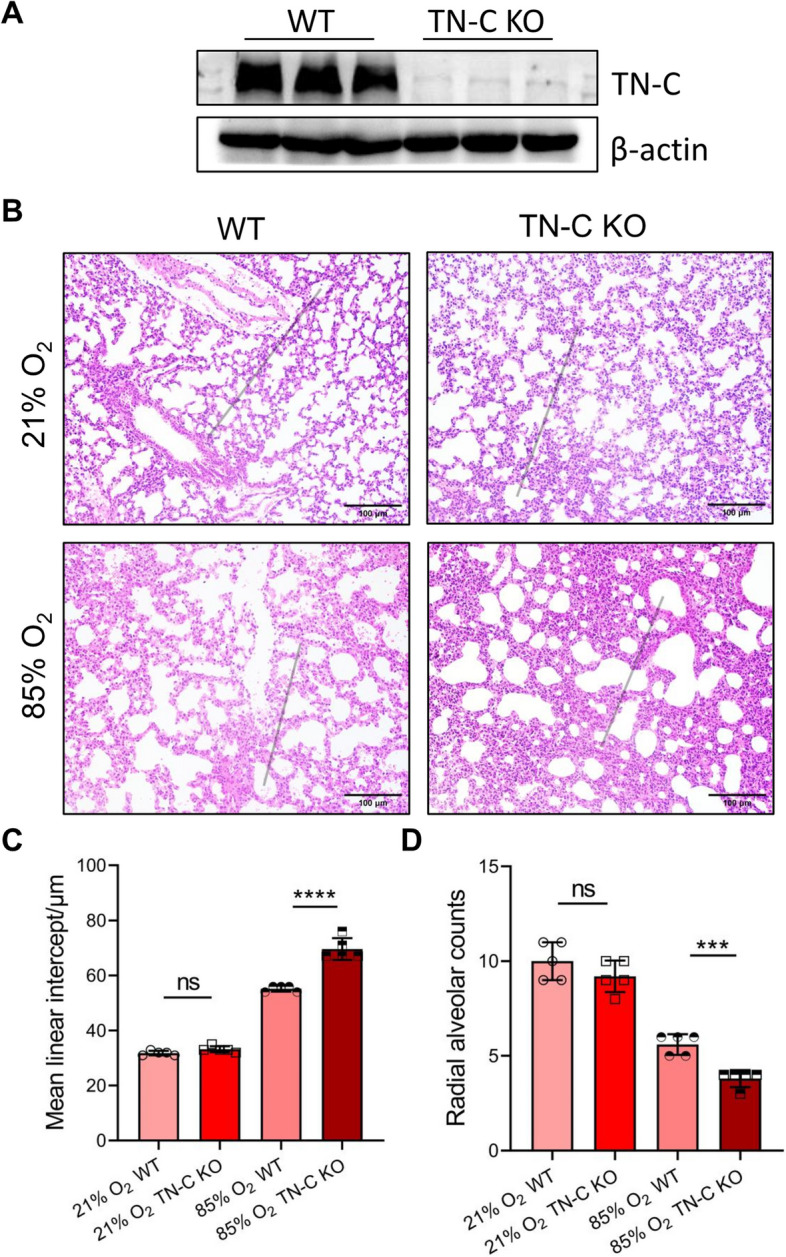
TN-C deficiency aggravates the disease severity of BPD. To establish the BPD model, newborn mice were exposed to 85% oxygen from postnatal day (P) 1 to P7, after which the mice were placed under normoxic conditions until P14. **A** Lung TN-C expression was abolished in knockout mice via Western blotting analysis. **B** Representative H&E-stained lung sections at P14. Scale bar = 100 μm. **C**-**D** The TN-C KO mice exhibited a lower RAC and a greater MLI than did the wild-type mice reared under hyperoxia from birth to P7

To provide direct genetic evidence that disrupting TN-C may affect BPD development, we established a mouse model of BPD using TN-C-knockout mice. Western blotting was used to identify the absence of TN-C protein in the lung of TN-C knockout mice (Fig. 6A). As shown in Fig. 6B, histological analysis on day 14 post birth showed that TN-C deficiency did not significantly affect normally developing alveolar architecture, which was in line with the findings of previous reports [17]. Moreover, TN-C deficiency delayed alveolar development in BPD-like mice, as indicated by a decreased number of alveoli and thickened alveolar septa (Fig. 6C-D). We speculated that, in line with in vitro findings, sTN-C promoted alveolar epithelial cell proliferation and migration and that deficiency of TN-C worsened BPD disease severity.

### TN-C plays a dual role in alveolarization

**Fig. 7:**
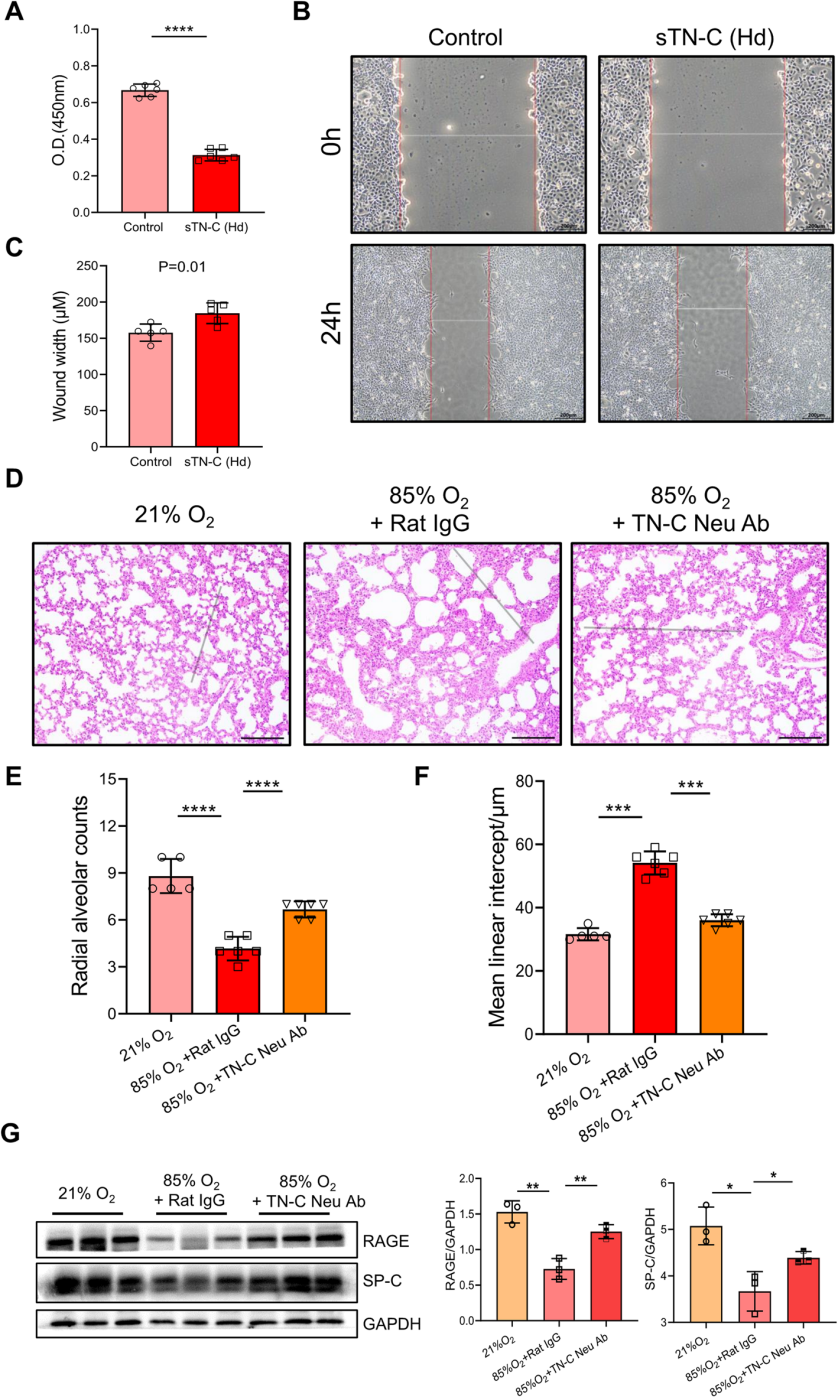
Excess TN-C slowed alveolar formation. **A** CCK-8 assay for determining the viability of MLE-12 cells incubated for 24 h under normoxia after low-dose sTN-C supplementation (8 ng/mL). **B**-**C** The migration ability of alveolar epithelial cells was measured by the scratch method. In a mouse model of BPD, newborn mice at P2, P4, P6, P8, and P10 received continual intraperitoneal injections of isotype rat IgG antibodies or TN-C neutralizing antibodies at a concentration of 1 μg/body. **D**-**F** Lung morphometry was analyzed via H&E staining of tissue samples from surviving pups at P14. Scale bar = 100 μm. **G** RAGE and SP-C protein expression in lung tissues. *P < 0.05, ** *P* < 0.01, ***P < 0.001, **** *P* < 0.0001

Previous results have demonstrated the supportive role of low-dose sTN-C in alveolar development in BPD patients. Given that the TN-C protein level was abnormally elevated in the lungs of BPD-like mice, we hypothesized that TN-C has a bidirectional effect on the development of BPD, as observed for fibronectin and other ECM proteins. We used a higher dose of sTN-C (8 ng/mL) to stimulate MLE-12 cells and found that both cell proliferation and migration were inhibited (Fig. 7A-C). Next, we determined whether anti-TN-C therapy could reverse impaired alveolar development in BPD patients by treating mice during postnatal alveolar maturation from P1 to P14. As measured by histological evaluation, treatment with TN-C neutralizing antibodies ameliorated the impairment of alveolar development in BPD-like mice, as indicated by an increased number of alveoli and a decreased mean linear intercept (Fig. 7D-F).

We also detected the level of AT1 marker RAGE and AT2 marker SP-C, and found that anti-TN-C therapy partially recovered the expression of RAGE and SP-C in BPD-like mice (Fig. 7G), implying that TN-C may be involved with AT1/AT2 cell differentiation. Overall, TN-C is a dual-edged sword in the lung development and pathogenesis of BPD.

## Discussion

BPD is the most common complication in preterm infants and occurs in up to 45% of infants born at gestational age < 29 weeks [2]. The lung development of premature infants is still immature (at the canalicular or saccular stage) [34], and neonatal respiratory support at delivery or postnatal inflammatory exposure can easily injure the premature lung and inhibit the development of alveoli and capillaries, thereby increasing the risk of BPD [35]. The lung development of neonatal mice resembled that of human infants at a gestational age of 24–38 weeks, and these mice were exposed to 85% hyperoxia for 7 consecutive days to mimic the clinical BPD lung phenotype [36, 37].

Previously, we found that the ECM protein fibronectin is involved in alveolar maturation and therefore contributes to BPD development [7, 38]. The synthesis and maturation of the alveolar epithelial ECM is an important step in alveolar formation [39]. Accordingly, we used proteomics to analyze the dynamic changes in ECM proteins and components during alveolar development in neonatal mice. TN-C protein expression first increased (P1-P7) but subsequently decreased (P7-P14) during alveolarization in postnatal mice. We speculated that the first increase in TN-C may help in alveolar differentiation, and the decreased demand for TN-C after alveolar maturation leads to a decrease in the TN-C protein, similar to the scaffolding used to construct a building, which must be removed after completion. Our present study demonstrated an elevated level of TN-C protein in lung tissue in a mouse model of BPD stimulated with 85% oxygen. The lungs of neonatal mice are still immature, and alveolarization is arrested under hyperoxia, which mainly manifests as larger, simplified, cystic alveoli and irregular pulmonary vessels [5].

On postnatal day 14, the TN-C signal was more pronounced and concentrated in the tips of injured alveolar septa under hyperoxia than under normoxia. In this work, we focused on the soluble form of the TN-C protein in a hyperoxia-treated cell model. As previously mentioned, cultured lung alveolar cells only express very little total TN-C [40]. We previously reported that HIF-1α mediates the migration of alveolar epithelial cells and participates in the progression of BPD [41]. In the present study, we demonstrated that pulmonary epithelial cells could release more soluble TN-C in response to hyperoxia, a phenomenon that could be connected to the HIF-1α pathway.

Our data demonstrated that low-dose sTN-C could promote the proliferation and migration of alveolar epithelial cells. A reduction in cell proliferation was observed in the hyperoxia-treated group when neutralizing antibodies against TN-C were utilized to disrupt a portion of the function of sTN-C. Additionally, alveolar epithelial cells exhibited enhanced proliferation and migration when low-dose sTN-C was administered directly under normoxic conditions. Simultaneously, newborn TN-C-knockout mice exhibited aggravated alveolar growth arrest after stimulation with 85% oxygen. On the other hand, our findings were consistent with those of a previous report [17], suggesting that there were no appreciable alterations in septa or alveoli between TN-C-knockout and wild-type lungs at P14 in a normoxic environment. Based on the above findings, we concluded that hyperoxia-induced lung injury may increase sTN-C through HIF-1α to ameliorate lung development. Mechanistically, soluble TN-C promoted the proliferation and migration of alveolar epithelial cells, at least in part through the AGE-RAGE pathway. Advanced glycation end products (AGEs) increase ICAM-1 expression via RAGE and may exacerbate the progression of many diseases [42]. ICAM-1 accumulates in the arterial plasma of infants with BPD [43]. Since the expression of integrin β3 resembles that of ICAM-1 [44–46] or TN-C [47] in many diseases, we hypothesized that the TN-C produced and secreted by alveolar epithelial cells promotes type II pneumocyte proliferation and growth through an autocrine mechanism via integrin β3 in cooperation with ICAM-1.

On the other hand, we discovered that supplementing alveolar epithelial cells with additional excess TN-C protein in vitro inhibited cell proliferation and migration. In addition, functional blockade with anti-TN-C antibodies improved alveolar development arrest in BPD-like mice in our study. However, excessive TN-C protein limited alveolar formation in BPD-like mice. According to previous literature, in a hyperoxia (85% O_2_)-based mouse model of BPD, blunted alveolarization was accompanied by increased levels of the lung ECM component collagen, and the elastin fibers in the septa were disorganized from “point-like” to “feathery” in appearance [48]. Consequently, we hypothesized that excessive TN-C may inhibit the movement of secondary alveolar cristae, forcing the alveolar wall to further elongate to expand the gas exchange area, which results in the pathological features of BPD, namely, an enlarged alveolar area and thickened septa. Herein, hyperoxia facilitated sTN-C release but did not allow stable sTN-C release to boost alveolar epithelial cell development, suggesting that the balance of TN-C synthesis plays an important role in the progression of BPD.

Although these studies revealed important discoveries, they have several limitations. First, we lacked direct evidence of increased TN-C content in bronchoalveolar lavage fluid (BALF) from BPD infants due to the challenges of collecting clinical specimens from living infants with BPD. Indeed, the expression of TN-C in the lung tissue of BPD infants was significantly greater than that in the lung tissue of extremely preterm or term infants [49]. Second, the intracellular signaling mechanism through which sTN-C promotes alveolar epithelial cell proliferation has not been thoroughly investigated, and only RNA sequencing analysis revealed that the AGE-RAGE pathway was strongly correlated with this process. It has been reported that AGE binding to RAGE promotes inflammation by activating NF-κB [29], and RAGE is strongly associated with hyperoxia-mediated lung pathologies during alveolarization [30]. We focused on epithelial cells, which dominate lung morphogenesis. TN-C interacts not only with epithelial cells but also with other cells, i.e., macrophages and fibroblasts. The roles and mechanisms of TN-C in lung development and BPD warrant further research. Third, in terms of treating BPD, it may be possible to improve alveolar growth by eliminating excessive TN-C in the lungs of BPD infants. However, TN-C play a dual role in the lung development and BPD pathogenesis. The timing of targeting TN-C therapy against BPD would be a great challenge.

## Conclusion

Together, these data point toward important biological and pathological roles for TN-C in alveolar development and BPD pathogenesis. Moderate TN-C promotes alveolar development, whereas increased TN-C in pulmonary tissues from BPD-like mice may cause arrested alveolarization. A delicate balance of TN-C and other ECM components may be indispensable in BPD therapy.

## Methods

### Experimental animals and ethics statement

Pregnant C57BL/6 J mice at embryonic days 18–19 were obtained from the Laboratory Animal Center of Nanjing Medical University (Nanjing, China) and housed under specific pathogen-free conditions at Nanjing Medical University.

TN-C knockout mice on a C57BL/6 J background were obtained from Cyagen Biosciences (Suzhou, China). All animal treatments were in accordance with the guidelines approved by the Institutional Animal Care and Use Committee of Nanjing Medical University (*IACUC-1708004*).

### Proteomics assay

#### Lung sample collection

The workflow of the study is presented in Fig. 1A. Briefly, developing pups designated for proteomics were euthanized at postnatal day (P) 1, 4, 7 or 14 by 200 mg/kg intraperitoneal (i.p.) injection of pentobarbital sodium. After aortic transection, a thoracotomy was performed, and the lungs were removed, dissected into individual lobes, and shortly rinsed with Dulbecco’s PBS (DPBS, Servicebio, Wuhan, China). The lung tissue samples were collected and snap-frozen in liquid nitrogen before being stored at -80 °C until needed.

#### Protein digestion and TMT tag labeling

The lung tissues were homogenized in RIPA working solution and subsequently centrifuged at 12,000 rpm for 10 min at 4 °C. The protein concentrations of the samples were determined by an Enhanced BCA Protein Assay Kit (Beyotime, Shanghai, China). To generate the peptides, 50 μg of protein was digested with 1 μg of trypsin (Promega, Madison, WI, USA) overnight at 37 °C. The peptides were subsequently labeled with TMT isobaric tags (Thermo Scientific, Rockford, USA) at RT for 1 h.

#### High-pH prefractionation

The labeled peptides were mixed into one component in equal amounts, and after desalting, the lyophilized peptide samples were reconstituted to 50 μl with mobile phase A (10 mM ammonium acetate aqueous solution, pH = 10) and separated under alkaline conditions using a C18 analytical column (XBridge BEH C18 XP Column, USA). The following gradient conditions were used: a liquid phase gradient of 60 min, mobile phase B (10 mM ammonium acetate; Sigma–Aldrich, St. Louis, MO, USA), 10% H_2_O, 90% acetonitrile (ANPEL Laboratory Technologies, Shanghai, China), and pH = 10.), 5% for 2 min, 5–30% for 40 min, 30-40% for 10 min, 40-90% for 4 min, 90% for 2 min, and 2% for 2 min. One component was collected every 1 min, collected in cycles, combined into 12 components, and stored at -80 °C after vacuum drying.

#### Nano-LC–MS/MS analysis

For each sample, 2 μg of total peptide was separated and analyzed with an UltiMate 3000 coupled to a Q Exactive HFX Orbitrap instrument (Thermo Fisher Scientific, USA) with a nanoelectrospray ionization source. Separation was performed using a reversed-phase column (Reprosil-Pur 120 C18-AQ, Dr. Maisch, Germany). The mobile phases used were H_2_O with 0.1% formic acid, 2% acetonitrile (phase A) and H_2_O with 0.1% formic acid and 80% acetonitrile (phase B). Separation of the sample was executed with a 90 min effective gradient at a 300 nL/min flow rate, and 0–10 min of sample loading was performed. Dependent acquisition (DDA) was performed in profile and positive mode with an Orbitrap analyzer at a resolution of 120,000 (@200 m/z) and a m/z range of 350–1600 for MS1; For MS2, the resolution was set to 45 k with a fixed first mass of 110 m/z. The automatic gain control target for MS1 was set to 3E6 with a maximum IT of 30 ms, and that for MS2 was set to 1E5 with a maximum IT of 96 ms. The top 20 most intense ions were fragmented by HCD with a normalized collision energy (NCE) of 32% and an isolation window of 0.7 m/z. The dynamic exclusion time window was 45 s, and single-charged peaks and peaks with charges exceeding 6 were excluded from the DDA [50].

#### Database search and quantification

The raw data files were searched and analyzed by Proteome Discoverer software (version 2.4.0.305; Thermo Fisher Scientific) and the built-in Sequest HT search engine. MS spectra lists were searched against their species-level UniProt FASTA databases (UniProt-Mus + musculus-10090-2021-8.fasta), with carbamidomethyl [C], TMT Pro (K) and TMT Pro (N-term) as fixed modifications and Oxi-dation (M) and acetyl (protein N-term) as variable modifications. Trypsin was used as a protease. A maximum of 2 missed cleavages was allowed. The false discovery rate (FDR) was set to 0.01 for both the PSM and peptide levels. Peptide identification was performed with an initial precursor mass deviation of up to 10 ppm and a fragment mass deviation of 0.02 Da. Unique peptides and Razor peptides were used for protein quantification, and the total peptide concentration was used for normalization. All the other parameters were set to their defaults.

#### Bioinformatics analysis

The original data contained 12 experimental samples. After screening for the number of unique peptides, proteins with a unique peptide number greater than or equal to 1 were retained, and 7660 proteins were retained after pretreatment. The data were logarithmically and centrally processed using R (version 3.6.3) or SIMCA software (version 16.0.2; Sartorius Stedim Data Analytics AB, Umea, Sweden). Statistical methods were used to screen differentially expressed proteins, and adjusted *P* values < 0.05 and fold change ≤ 0.67 or fold change ≥ 1.5 were considered to indicate statistical significance [51]. In this project, the Kyoko Encyclopedia of Genes and Genomes (KEGG) and pathway databases (www.kegg.jp/kegg/pathway.html) were used to search the *Mus musculus* (mouse) database. The significantly enriched metabolic pathways of the differentially expressed ECM proteins were also analyzed. K-means clustering was subsequently performed in combination with heatmaps to visualize the dynamic changes in ECM components related to lung development in neonatal mice.

### Animal model of BPD

The murine model of BPD was established as described previously [52, 53]. Briefly, newborn mouse pups from several litters were mixed and divided into equal-sized cages of 6-8. Cages were then maintained in either indoor air (21% oxygen) or 85% oxygen within 12 h after birth until the day of harvest. The temperature (22 °C) and humidity (50-60%) were kept constant. To avoid oxygen toxicity in the dams and to eliminate maternal effects between groups, the nursing dams were rotated between the normoxic and hyperoxic groups every 48 h. All mice were maintained on a 12-h light–dark cycle. Mice were euthanized at P7 or P4 by injection of pentobarbital sodium (200 mg/kg i.p.).

To explore the role of TN-C in BPD pathogenesis, we treated TN-C-knockout pups with 85% oxygen to establish a BPD model. Wild-type BPD mice were used as the control group.

On postnatal days 2, 4, 6, 8 and 10, the mice were injected intraperitoneally with TN-C neutralizing antibodies or isotype rat IgG at 1 μg/g body weight in 10 μl of PBS. The mice were divided into three study groups: normoxic, hyperoxia + isotype and hyperoxia + TN-C neutralizing antibody.

### H&E imaging

The pups were sacrificed by intraperitoneal injection of pentobarbital sodium. After being euthanized, the mice were tracheotomized, and the right lungs were removed and snap frozen for subsequent experiments. The left lungs were inflated and fixed with 4% paraformaldehyde (Servicebio, Wuhan, China) at a pressure of 25 cm H_2_O for ≥ 15 min. After isolation, the left lungs were fixed in 4% formalin at 4 °C overnight. Paraffin-embedded lung tissue blocks were sectioned at 5 μm and stained with hematoxylin and eosin (H&E) stain using standard staining procedures on the pathology platform of Servicebio Technology (Wuhan, China). Five randomly selected areas from 5 μm H&E-stained lung sections were captured at × 200 magnification with a microscope (model BX-53, Olympus Optical) under identical lighting conditions and optical settings by an investigator blinded to the grouping. Image analysis was performed using research-based digital image analysis software (ImageJ, JAVA). Radial alveolar counts (RACs) were measured by standard morphological techniques [54]. Briefly, respiratory bronchioles were identified as bronchioles lined by epithelium in one part of the wall. From the center of the respiratory bronchiole, a perpendicular line was dropped to the edge of the acinus (connective tissues or septum or pleura), and the number of septae intersected by this line was counted. The mean linear intercept (MLI), an indicator of mean alveolar diameter, was determined by superimposing a predetermined grid on the image, setting randomly placed lines, and counting the number of times the lines crossed an air-tissue interface [55].

### Western blotting analysis

Total protein was extracted from cells or tissues by lysis with RIPA buffer containing protease and phosphatase inhibitor cocktails (Beyotime, Shanghai, China), after which the mixture was sonicated on ice 3 times for 20 s each. Protein concentrations were determined with a bicinchoninic acid (BCA) assay. Equal amounts of proteins were separated via SDS–PAGE. The proteins were separated by 10% SDS–PAGE and transferred to polyvinylidene fluoride (PVDF) membranes (Millipore, Billerica, USA). The membranes were blocked for 1 h in 5% skim milk at room temperature and incubated at 4 °C overnight with the following primary antibodies: anti-TN-C (ab108930, Abcam), anti-HIF-1α (ab179483, Abcam), anti-β-actin (GB15003, Servicebio), anti-RAGE (ab216329, Abcam), anti-SFTPC (10774-1-AP, Proteintech) and anti-GAPDH (GB15004, Servicebio). The membranes were then washed three times with Tris-buffered saline containing Tween-20 (TBST) and incubated with horseradish peroxidase (HRP)-conjugated goat anti-rabbit IgG (EarthOx Life Sciences, CA, USA) or goat anti-mouse IgG (H + L) HRP (s0002, Affinity Biosciences) for 1 h at room temperature. The antibody–antigen complexes were detected with Immobilon Western Chemiluminescent HRP Substrate (Millipore, MA, USA) and visualized using the G:Box gel doc system (Syngene, UK). For proteins in lung tissues and cells, β-actin was used as the internal control. For proteins in the supernatant, Ponceau S (P0022; Beyotime, Shanghai, China) was used as the loading control. The density quantification analysis was performed using software Image J.

### Immunohistochemistry

Formaldehyde-fixed mouse lungs were dehydrated, paraffin-embedded, and sectioned (5 μm thickness). Antigen retrieval was performed in 10 mM citrate buffer (pH 6.0) in a pressure cooker for 10 min. Endogenous peroxidase activity was inhibited by adding 3% H_2_O_2_ solution to the slides for 15 min, followed by a 30-min blocking step in which 3% BSA was added to the PBS. The slides were then incubated for 1 h at room temperature (RT) with a rabbit monoclonal TN-C antibody (1:200, ab108930, USA). After the samples were washed with PBS, secondary antibodies conjugated to horseradish peroxidase (HRP) were added to the samples, which were subsequently incubated for 50 min at RT. Then, freshly prepared DAB chromogenic reagent was added to mask the tissues. Finally, the samples were mounted with hematoxylin staining solution and scanned by a digital tissue section scanner (Pannoramic MIDI, 3DHistech, Hungary). Images of the slides were captured by CaseViewer 2.4 viewing software (3DHistech, Hungary).

### Cell culture and treatments

MLE-12 cells (CRL-2110, ATCC), a murine bronchial alveolar cell line, were cultured in DMEM (HyClone, USA), 10% fetal bovine serum (FBS, Lonsera, USA) and 1% penicillin/streptomycin (HyClone, USA). BEAS-2B cells (purchased from Procell Life Science & Technology Co. Ltd.), a kind of human bronchial epithelium, were cultured in complete bronchial epithelial cell growth medium (Lonza, CC-3170 and CC-4175). The cells were incubated in a humidified atmosphere of 5% CO_2_ at 37 °C. The cells were seeded in 24-well plates overnight and then exposed to room air (21% oxygen) or hyperoxia (85% oxygen) for 24 h. When needed, the HIF-1α inhibitor 2-MeOE2 (MCE, USA, S1233) or the HIF-1α agonist DMOG (MCE, USA, S7483) was added to the medium at a final concentration of 10 μM or 20 μM. The cells were harvested 12 h later for further analysis.

### Cell transfection

293 T cells (CRL-3216, ATCC), a human embryonic kidney cell line, were seeded and incubated overnight before transfection. After mixing with Liposomal Transfection Reagent (Yeasen, China) in DMEM without FBS, penicillin or streptomycin for 25 min, the expression plasmid encoding TN-C (Vector Builder, China) was then transfected into 293 T cells at 90-95% confluence in DMEM for 48 h. The cell supernatant was harvested for further analysis. The blank vehicle plasmid was used as a control.

### Cell proliferation and viability assay

Cell proliferation was detected by measuring active DNA synthesis using the Cell-Light™ EdU Apollo®567 Cell Tracking Kit (RiboBio, Guangzhou, China). The cells were seeded in 96-well plates at a density of 10000 cells/well and incubated overnight at 37 °C. Then, supernatant overexpressing the sTN-C protein and neutralizing TN-C antibody (MAB2138; R&D Systems, USA) were added to the culture media at concentrations of 2 or 8 ng/ml and 2 μg/ml, respectively. After incubating at 85% oxygen for 24 h, the fixed cells were treated with an EdU kit, and EdU incorporation was assessed via fluorescence. Changes in cell viability were determined by using the CCK-8 Cell Counting Kit (Vazyme Biotech Co., Ltd.). The absorbance values (OD450) were subsequently determined using a Multiscan microplate reader.

### Cell migration ability assay

When the MlE-12 cells seeded in 24-well plates reached confluence, a single scratch was made using a sterile yellow pipette tip. Then, the cells were incubated with the sTN-C protein at a concentration of 2 nglml (low dose) or 8 ng/mL (high dose). After incubation at 21% oxygen for 24 h, images of the scratches were captured by an Olympus IX73 inverted microscope at × 100 magnification. To further visualize cell migration, scratched MLE-12 cells treated with 2 ng/ml sTN-C protein in 24-well plates were transferred to a temperature- and CO_2_-controlled (37 °C, 5% CO_2_) environment with a Zeiss Cell Discoverer microscope system. Live-cell phase-gradient contrast images of the individual field regions inside each well were automatically acquired using ZEN Blue 2.3 software. Representative figure images were selected, and additional image postprocessing steps were performed in ImageJ.

### RNA extraction and high-throughput mRNA sequencing

After the cells were incubated with 2 ng/ml sTN-C protein (blank vector-transfected supernatant serving as a control) for 24 h, total RNA was isolated from the MLE-12 cells via a TRIzol reagent kit (Life Technologies, Carlsbad, CA, USA). To ensure the quality of the RNA, a Nanodrop was used to determine the purity of the RNA, and an Agilent 2100 was used to accurately determine the integrity of the RNA. When constructing the library, eukaryotic mRNA was first enriched with oligo (dT) magnetic beads, and fragmentation buffer was subsequently added to randomly interrupt the mRNA. Second, using mRNA as a template, the first cDNA strand was synthesized with six random primers, and then, buffer, dNTPs, RNase H and DNA polymerase I were added to synthesize the second cDNA strand. The cDNA was purified with AMPure XP beads. The purified double-stranded cDNA was then end repaired, added to the A tail and connected to the sequencing connector. Then, AMPure XP beans were used to determine the fragment size. Third, the cDNA library was obtained by PCR enrichment. After the construction of the library was completed, the library quality was tested, and computer sequencing was carried out only after the test results met the requirements. Finally, different libraries were pooled according to the target offline data volume and sequenced with the Illumina NovaSeq platform.

### Quantitative real-time PCR

Total RNA was extracted from fresh lung tissue or cells with a TRIzol reagent Kit (Vazyme, China) in accordance with the manufacturer’s instructions. The mRNAs were reverse transcribed with cDNA synthesis supermix for qRCR (11141, Yeasen). Quantitative real-time PCR (qRT–PCR) was performed with a StepOnePlus Real-Time PCR System (ABI, USA). The reaction mixture contained 5 μl of Universal Blue SYBR Master Mix (11184, Yeasen), 3 μl of RNase-free water, 0.5 μl of primer, and 1 μl of template. We used the fold change (2^−△△CT^) to show the expression of the mRNAs.

The sequences of primers used were as follows:

- mouse Icam1 forward, GTGATGCTCAGGTATCCATCCA
- mouse Icam1 reverse, CACAGTTCTCAAAGCACAGCG
- mouse β-actin forward, GAGAAGCTGTGCTATGTTGCT.
- mouse β-actin reverse, CTCCAGGGAGGAAGAGGATG.

### Statistical analysis

All the data were subjected to testing for a normal distribution. All the data are expressed as the mean ± SEM, and all the statistical analyses were performed using GraphPad Prism 8. Single comparisons were conducted by unpaired t tests. Multiple comparisons were tested by using one-way ANOVA with Tukey’s adjustment. Survival study comparisons were performed using Kaplan–Meier analysis. For all analyses, statistical significance was set as follows: *, *P* < 0.05; **, *P* < 0.05; ***, *P* < 0.01; ****, *P* < 0.001; and ns, not significant.

### Supplementary Information

**Additional file 1.** Live cell imaging system showing the migration capacity of alveolar epithelial cells at the scratch edge after a 90-min incubation period under normoxia following the addition of low-dose sTN-C (2 ng/mL).

**Additional file 2:** **Figure S1. **TN-C Immunofluorescence staining in mouse lungs. TN-C was diffusely expressed in lung tissue and the arrows indicated that abundant expression in bronchiolar epithelium. Scale bars = 100 μm. Additionally, TN-C was also expressed in alveolar epithelial cells as shown by co-staining with the type II alveolar epithelial cell marker SP-C. Squares represent areas enlarged in insets, Scale bars = 5 μm.

**Additional file 3.**

## Data Availability

The data are available from the corresponding author upon reasonable request.
