# Supplementary figures and images for: Tenascin-C modulates alveolarization in bronchopulmonary dysplasia
Source: Inflamm Regen. 2024 Mar 28;44:16. doi: 10.1186/s41232-024-00330-9 (PMC10976775; doi:10.1186/s41232-024-00330-9)

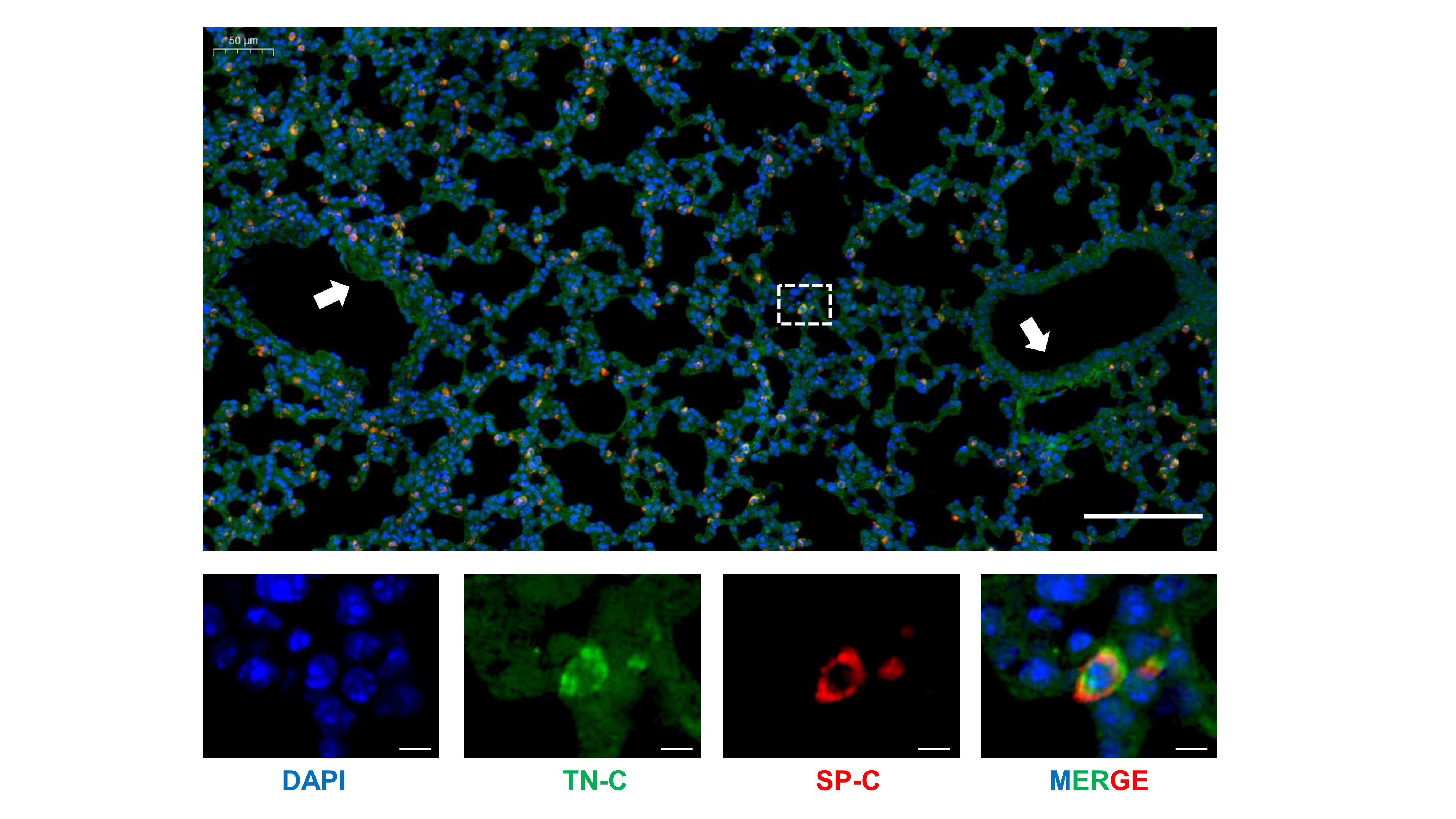

Supplement: Supplementary file 2 — Additional file 2: Figure S1. TN-C Immunofluorescence staining in mouse lungs. TN-C was diffusely expressed in lung tissue and the arrows indicated that abundant expression in bronchiolar epithelium. Scale bars = 100 μm. Additionally, TN-C was also expressed in alveolar epithelial cells as shown by co-staining with the type II alveolar epithelial cell marker SP-C. Squares represent areas enlarged in insets, Scale bars = 5 μm. [file 41232_2024_330_MOESM2_ESM.png]
