# Supplementary material for: Tenascin-C modulates alveolarization in bronchopulmonary dysplasia
Source: Inflamm Regen. 2024 Mar 28;44:16. doi: 10.1186/s41232-024-00330-9 (PMC10976775; doi:10.1186/s41232-024-00330-9)
Supplement: Supplementary file 3 — Additional file 3. [file 41232_2024_330_MOESM3_ESM.pptx]

## Slide 1
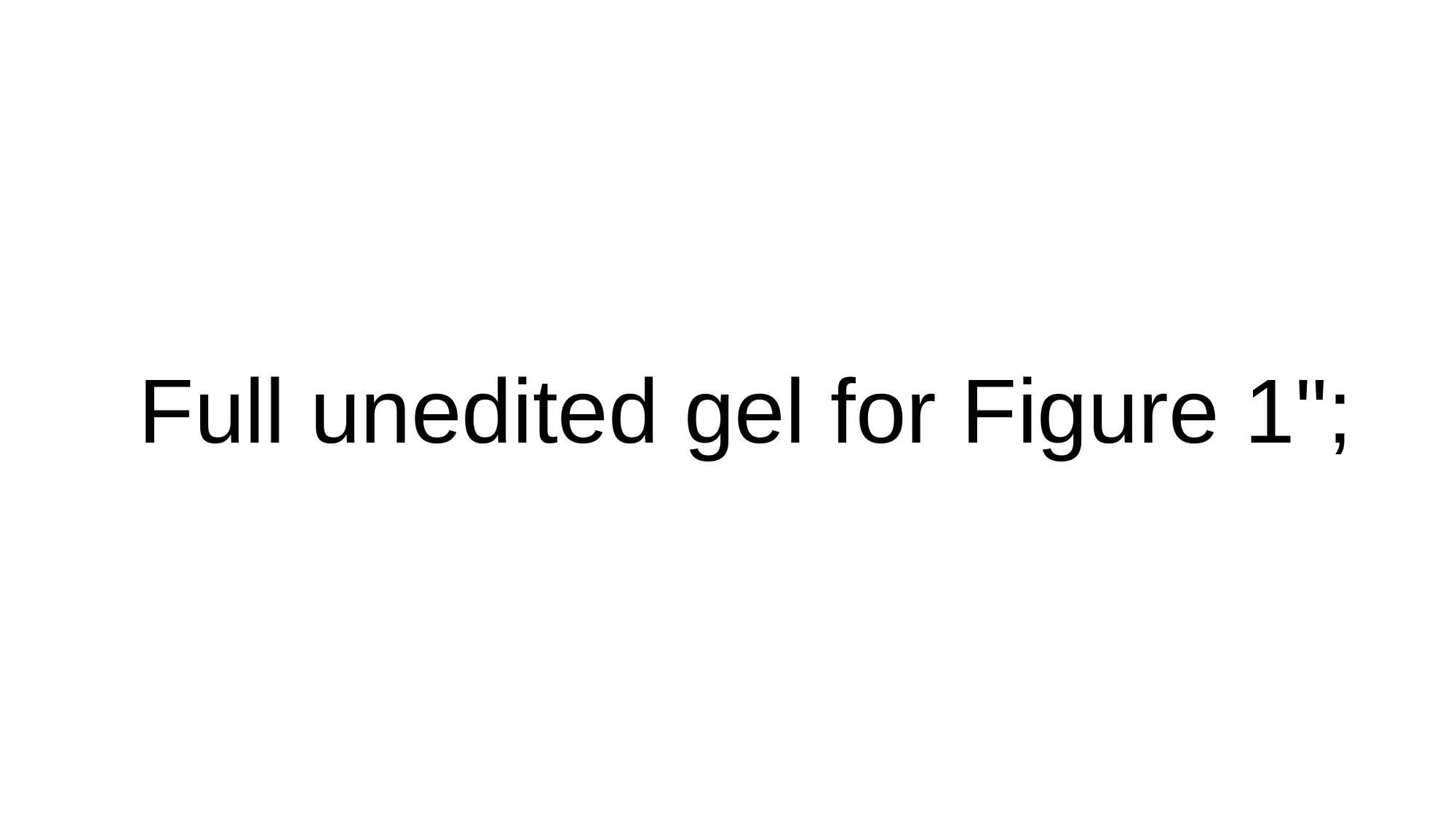

Full unedited gel for Figure 1";

## Slide 2
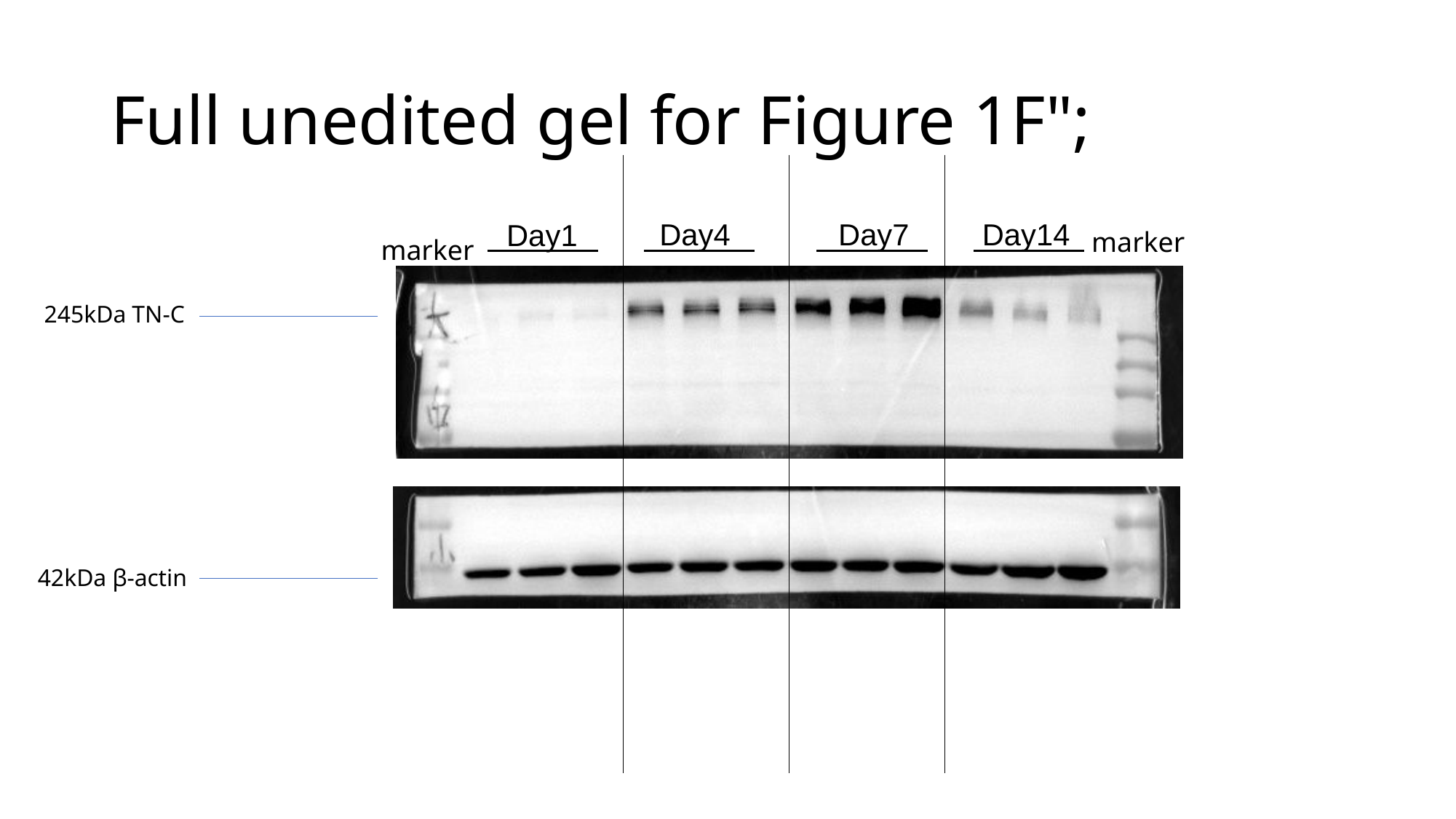

# Full unedited gel for Figure 1F";
Day7
Day4
Day14
Day1
marker
marker
245kDa TN-C
42kDa β-actin

## Slide 3
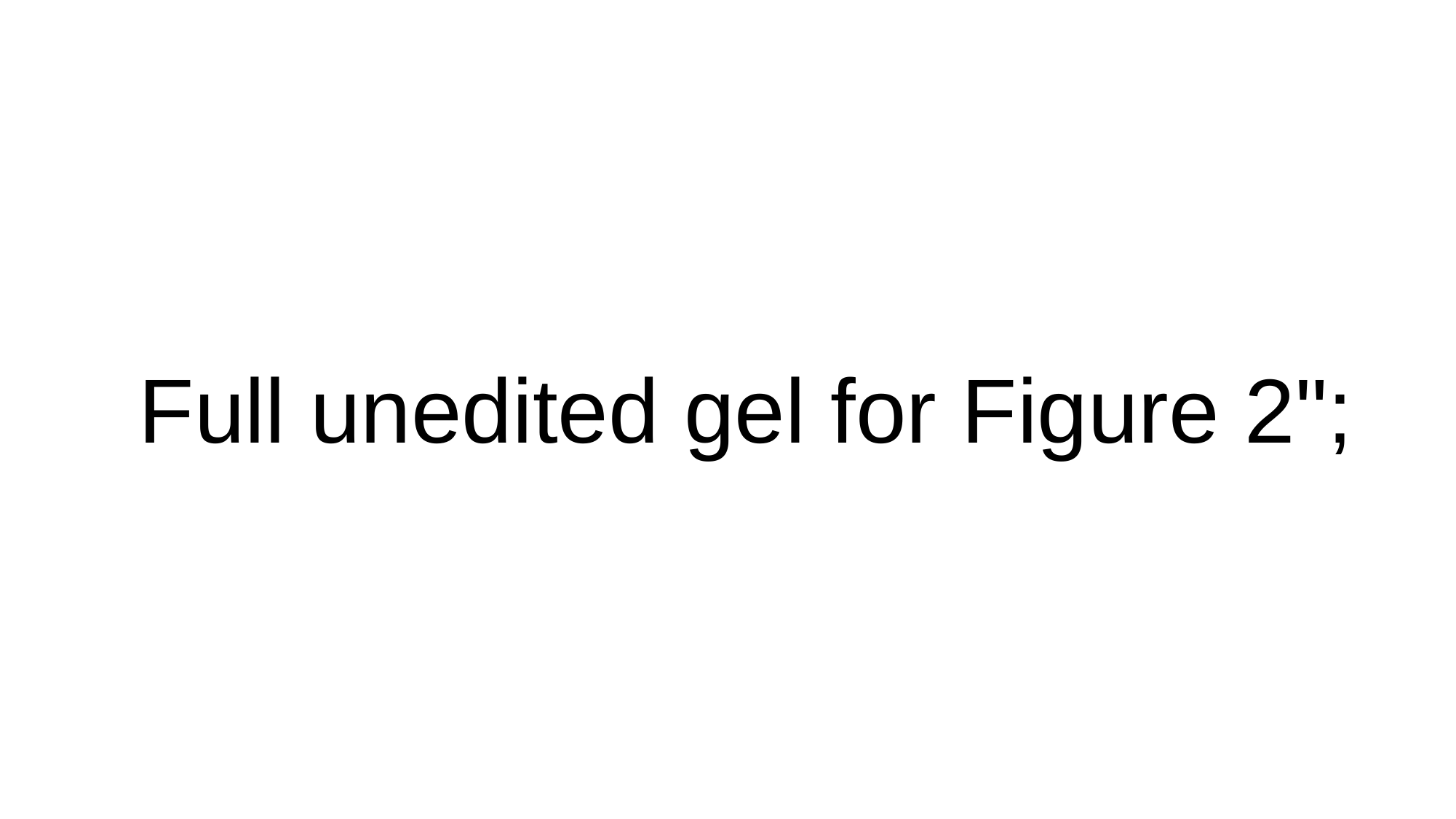

Full unedited gel for Figure 2";

## Slide 4
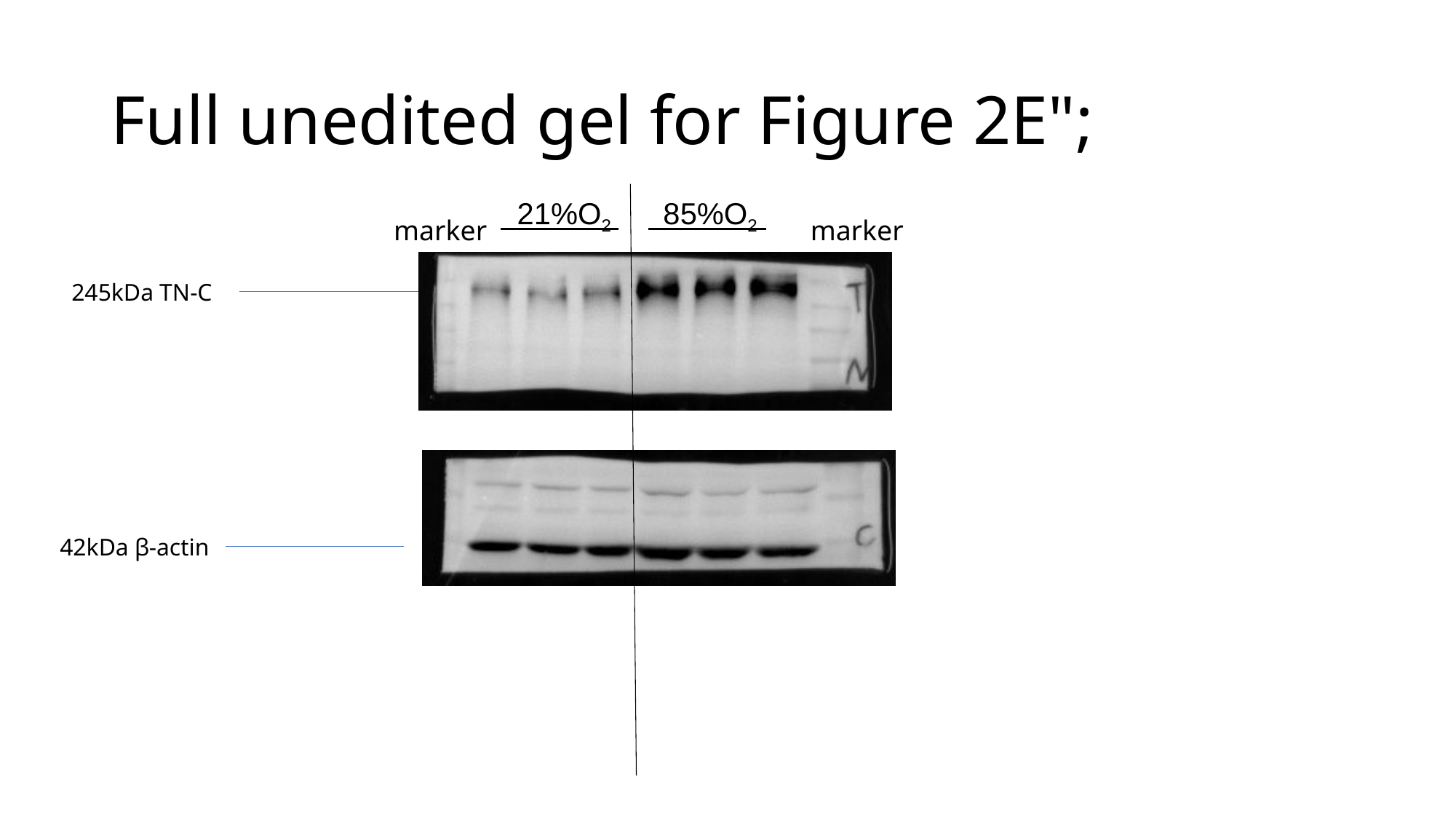

# Full unedited gel for Figure 2E";
21%O2
85%O2
marker
marker
245kDa TN-C
42kDa β-actin

## Slide 5
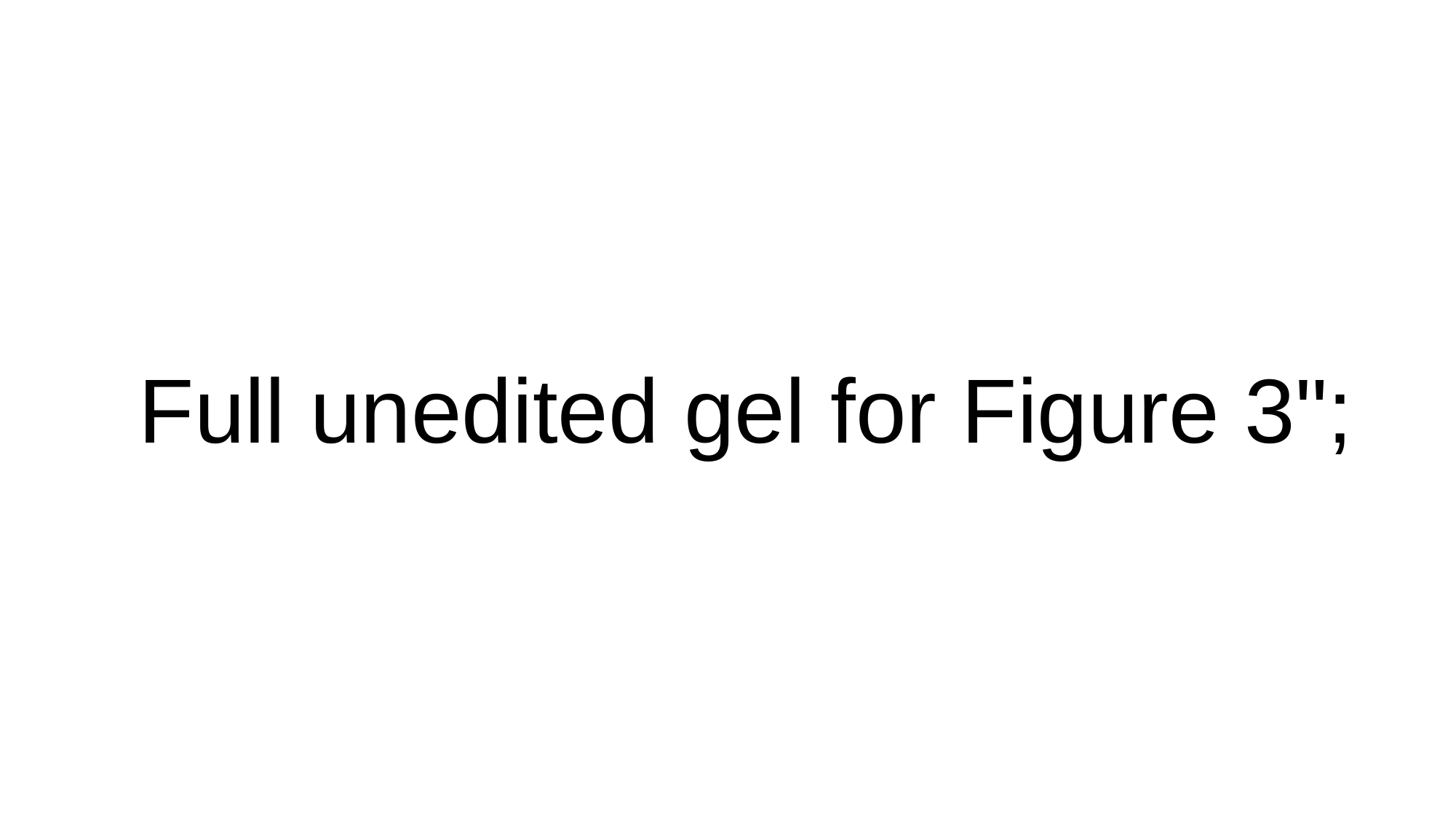

Full unedited gel for Figure 3";

## Slide 6
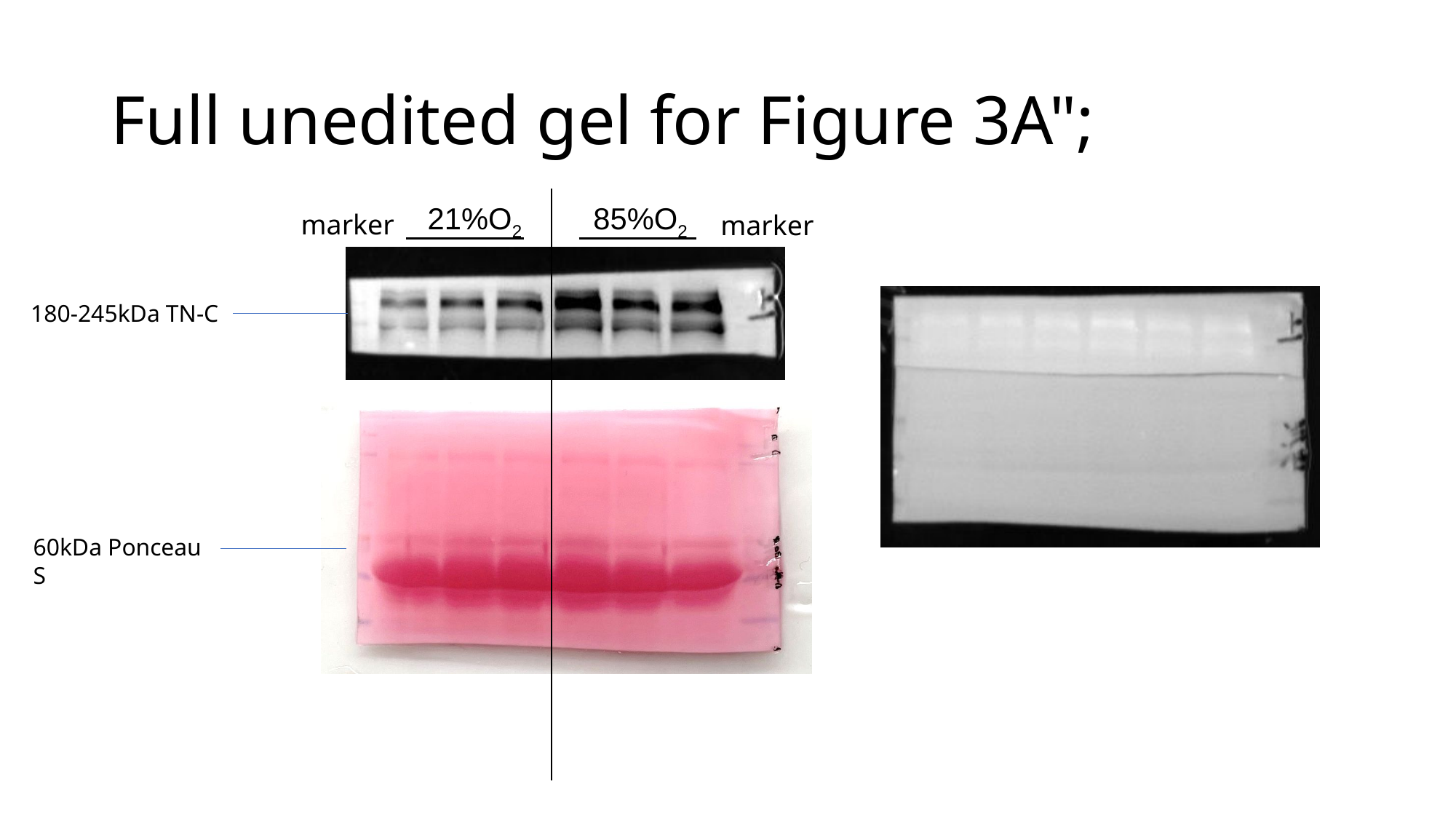

# Full unedited gel for Figure 3A";
21%O2
85%O2
marker
marker
180-245kDa TN-C
60kDa Ponceau S

## Slide 7
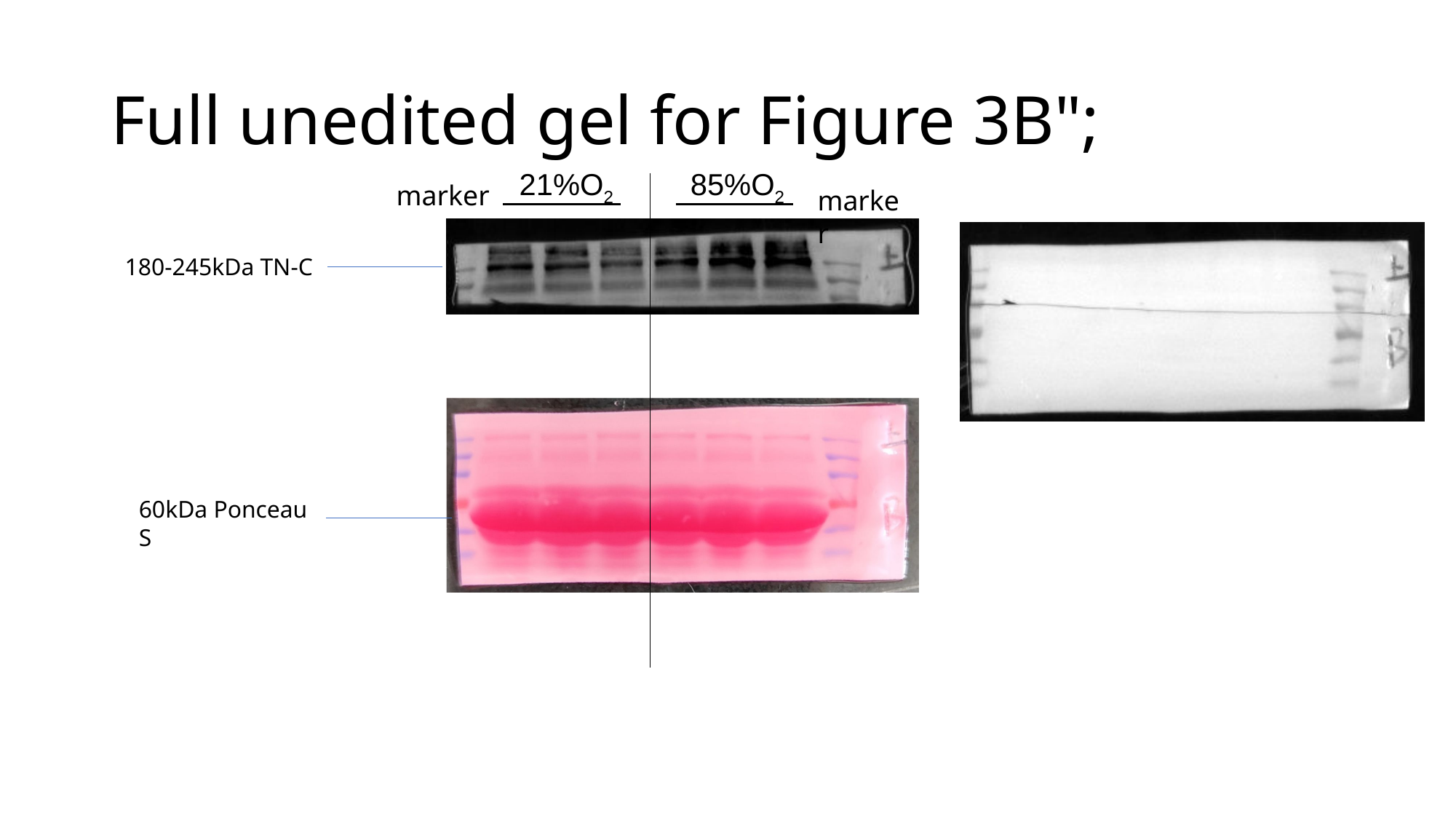

# Full unedited gel for Figure 3B";
21%O2
85%O2
marker
marker
180-245kDa TN-C
60kDa Ponceau S

## Slide 8
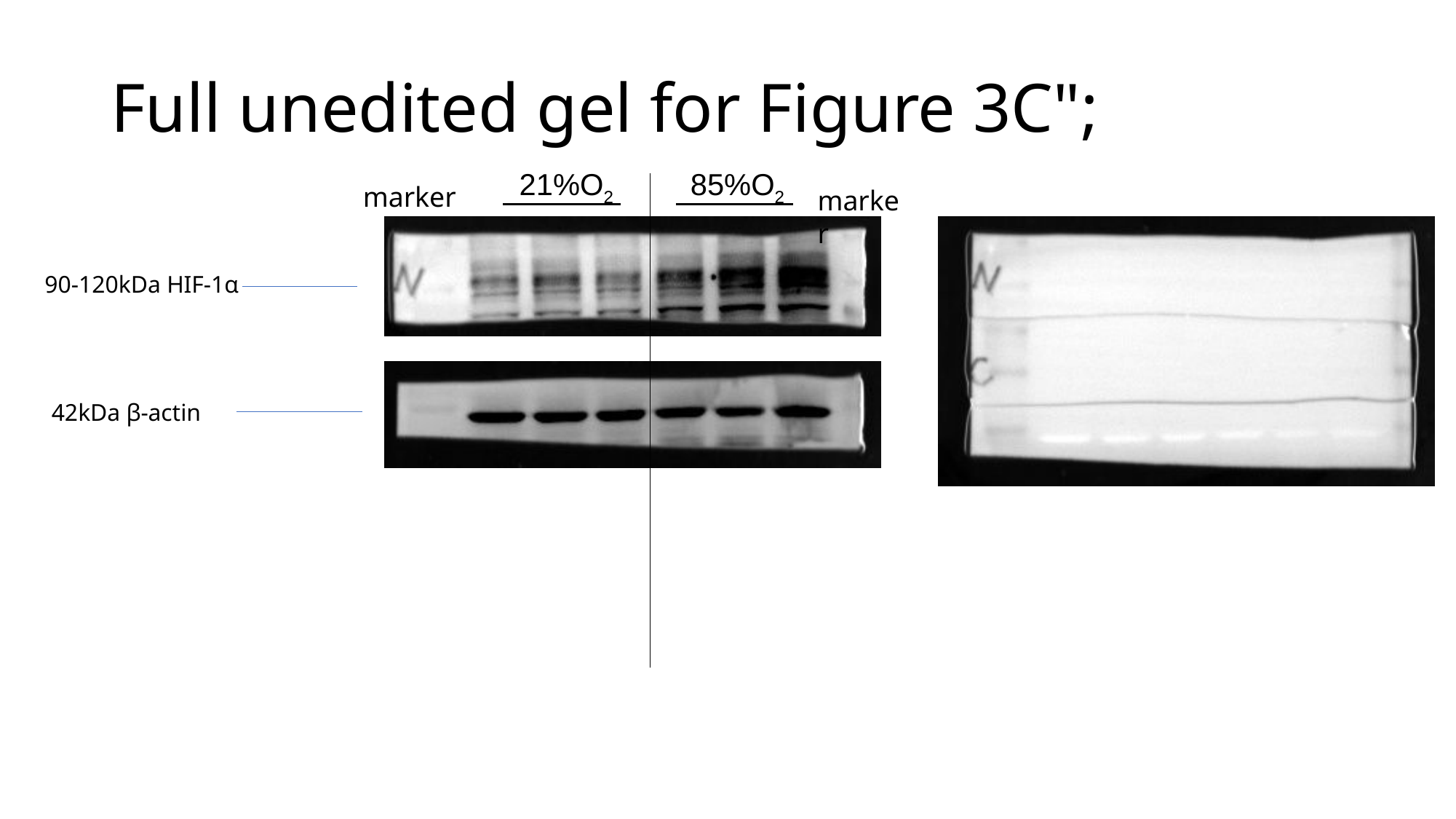

# Full unedited gel for Figure 3C";
21%O2
85%O2
marker
marker
90-120kDa HIF-1α
42kDa β-actin

## Slide 9
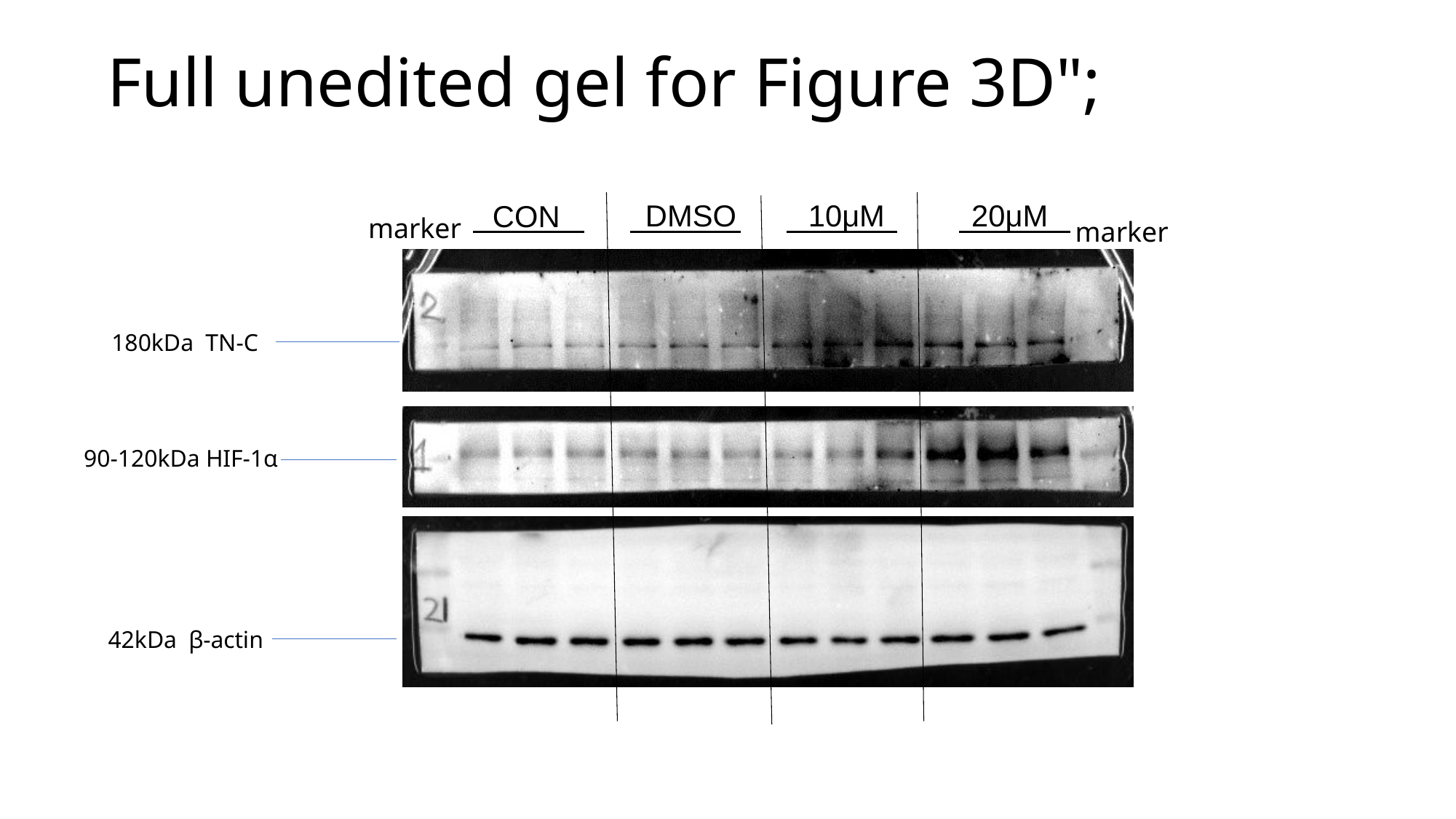

# Full unedited gel for Figure 3D";
10μM
20μM
DMSO
CON
marker
marker
180kDa TN-C
90-120kDa HIF-1α
42kDa β-actin

## Slide 10
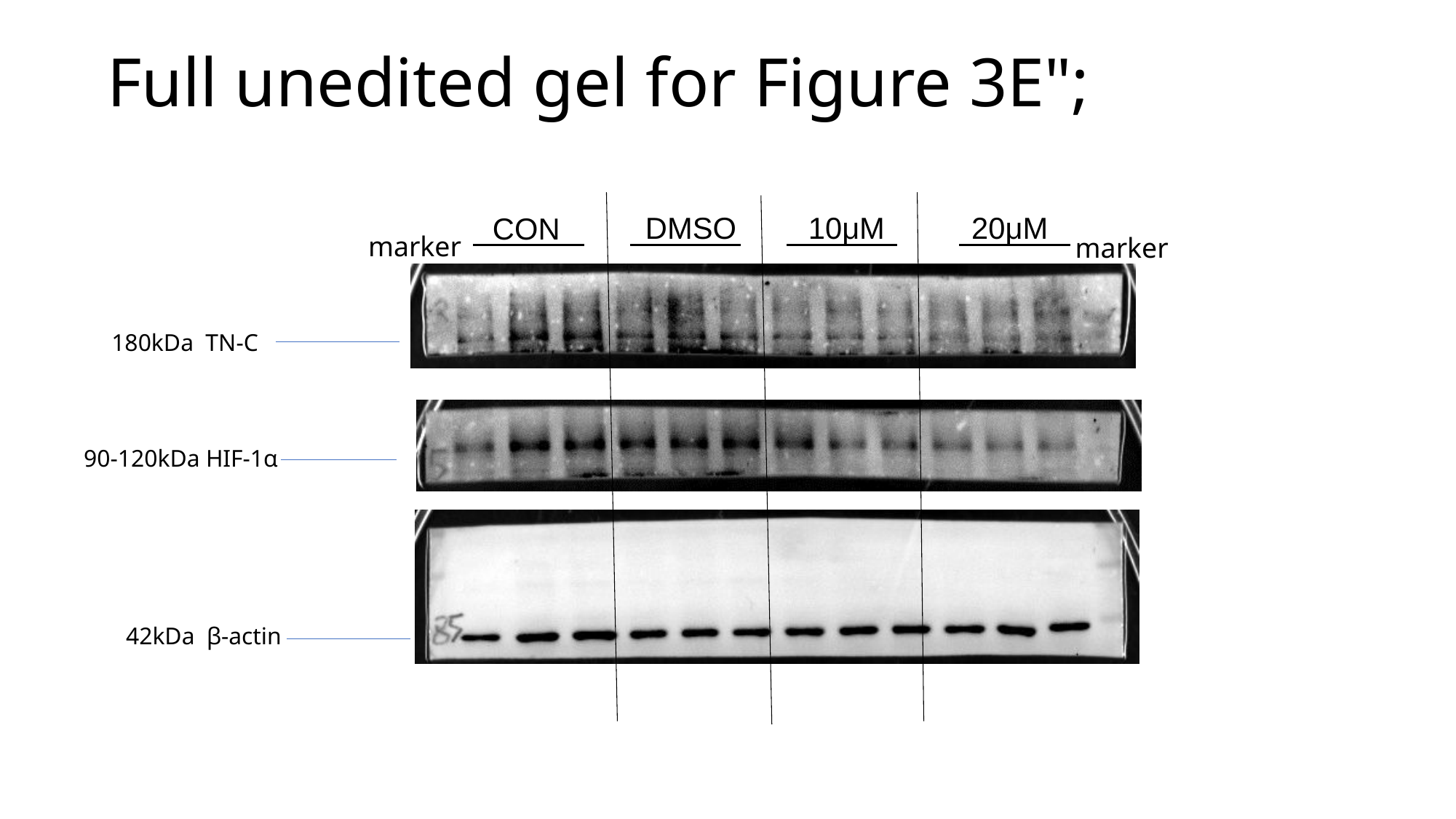

# Full unedited gel for Figure 3E";
10μM
20μM
DMSO
CON
marker
marker
180kDa TN-C
90-120kDa HIF-1α
42kDa β-actin

## Slide 11
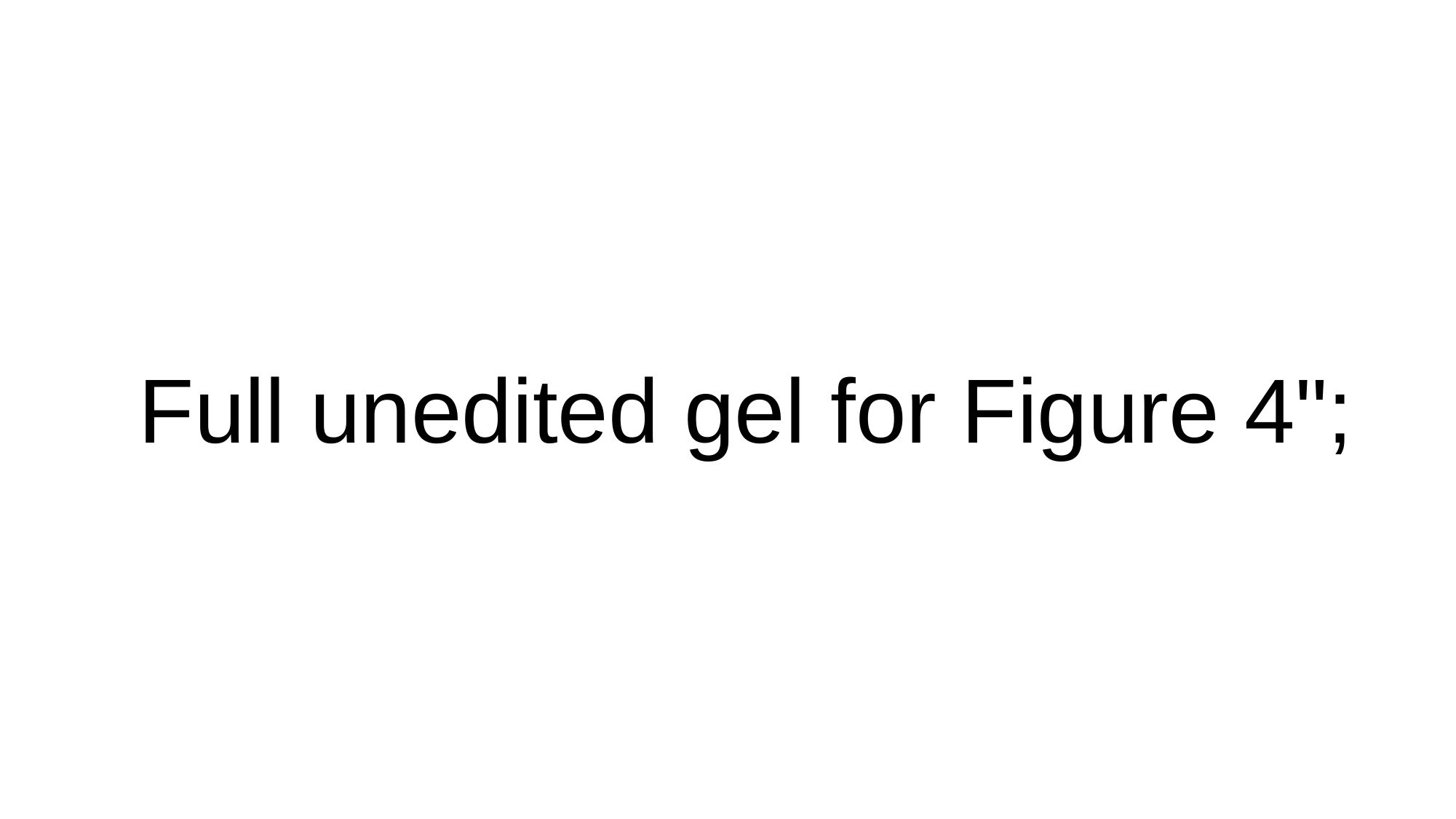

Full unedited gel for Figure 4";

## Slide 12
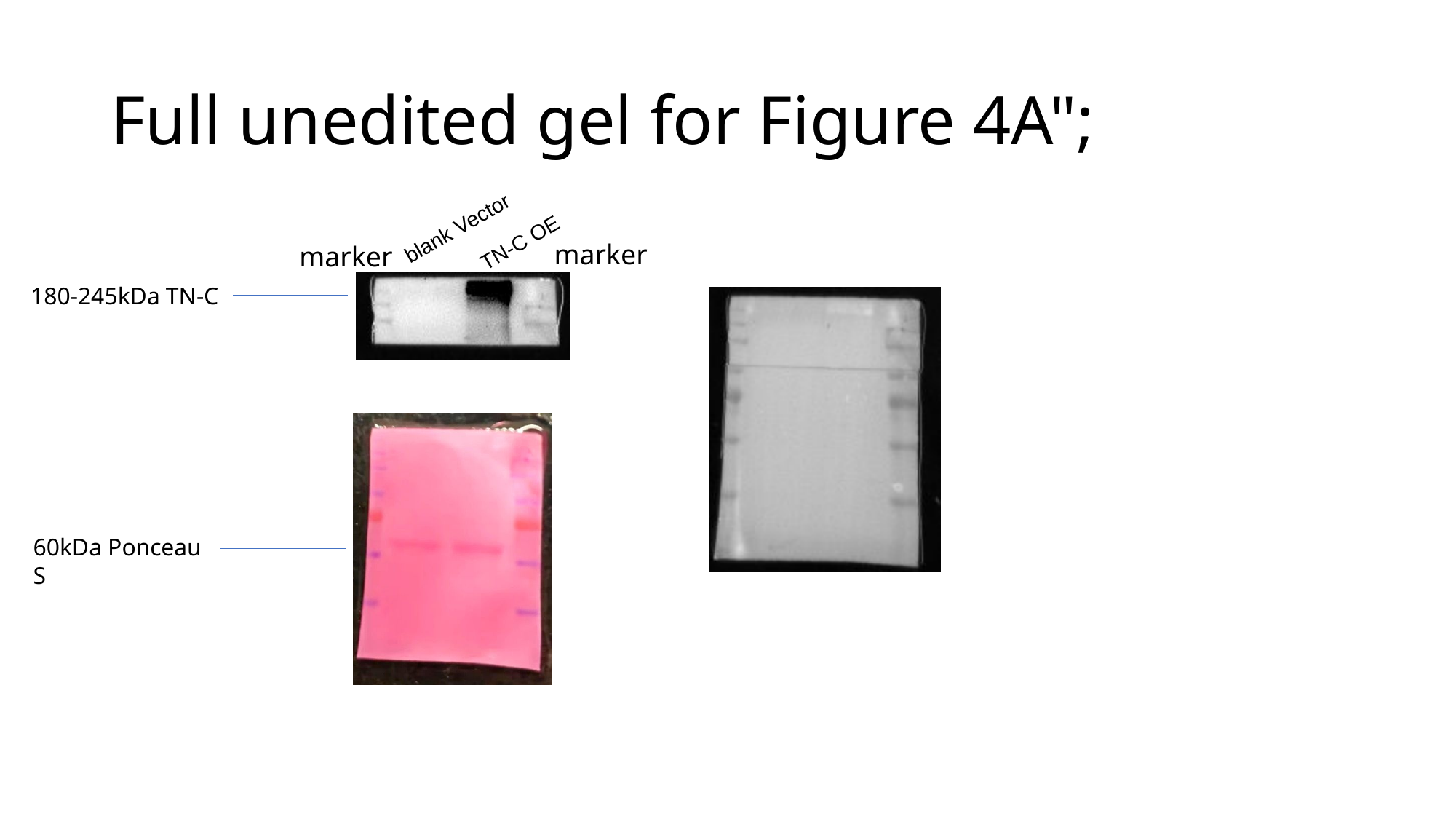

# Full unedited gel for Figure 4A";
blank Vector
TN-C OE
marker
marker
180-245kDa TN-C
60kDa Ponceau S

## Slide 13
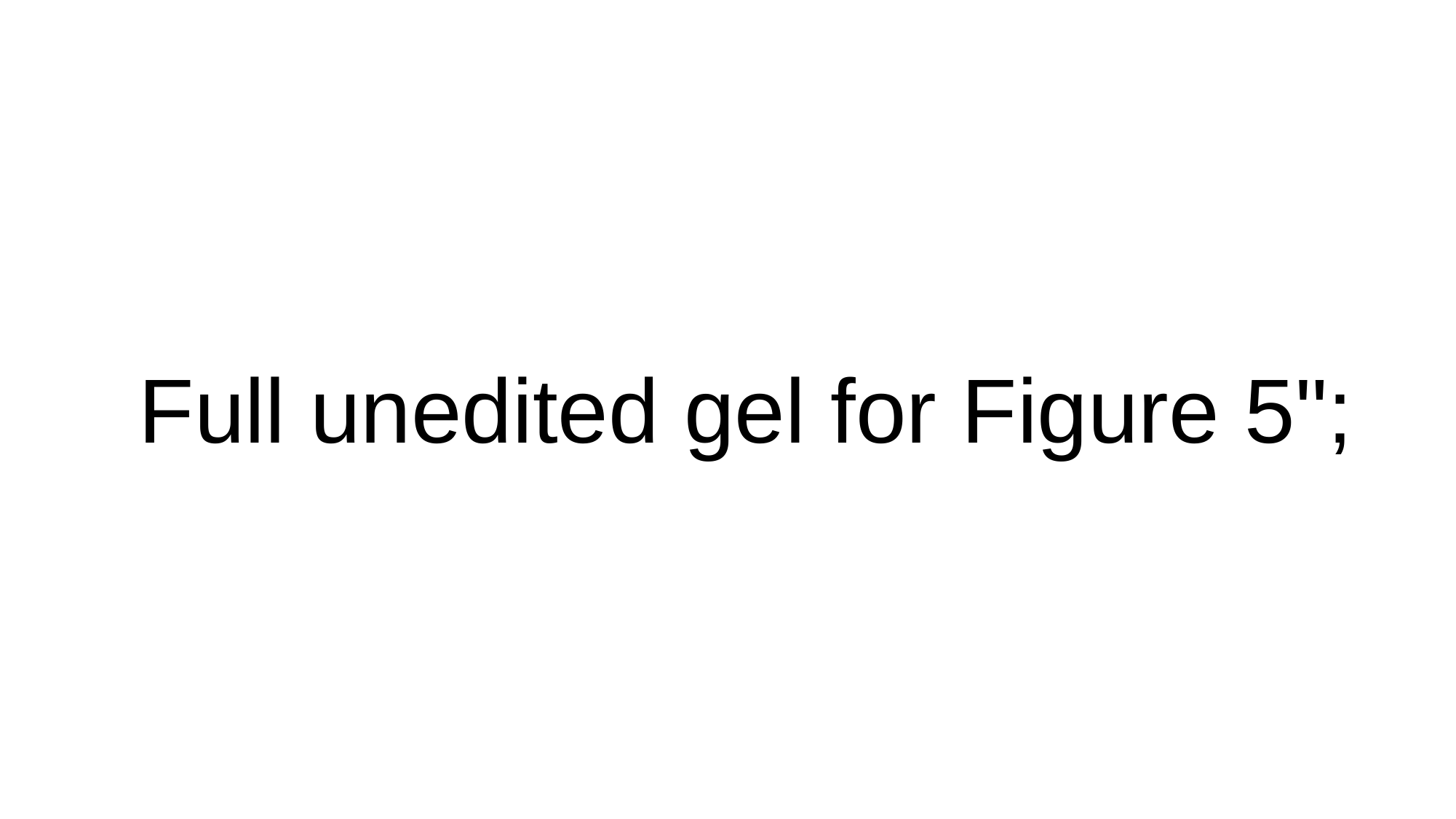

Full unedited gel for Figure 5";

## Slide 14
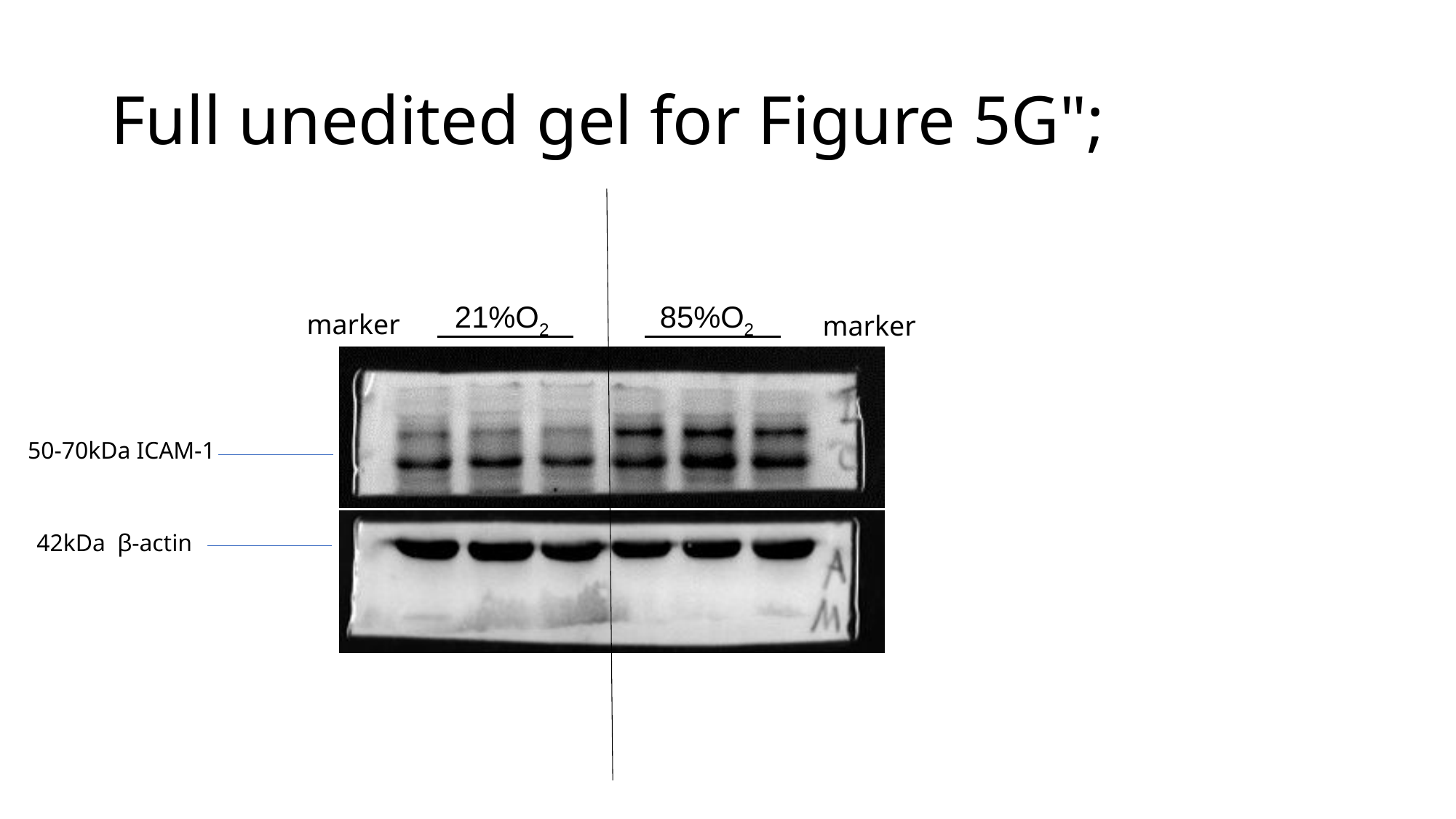

# Full unedited gel for Figure 5G";
21%O2
85%O2
marker
marker
50-70kDa ICAM-1
42kDa β-actin

## Slide 15
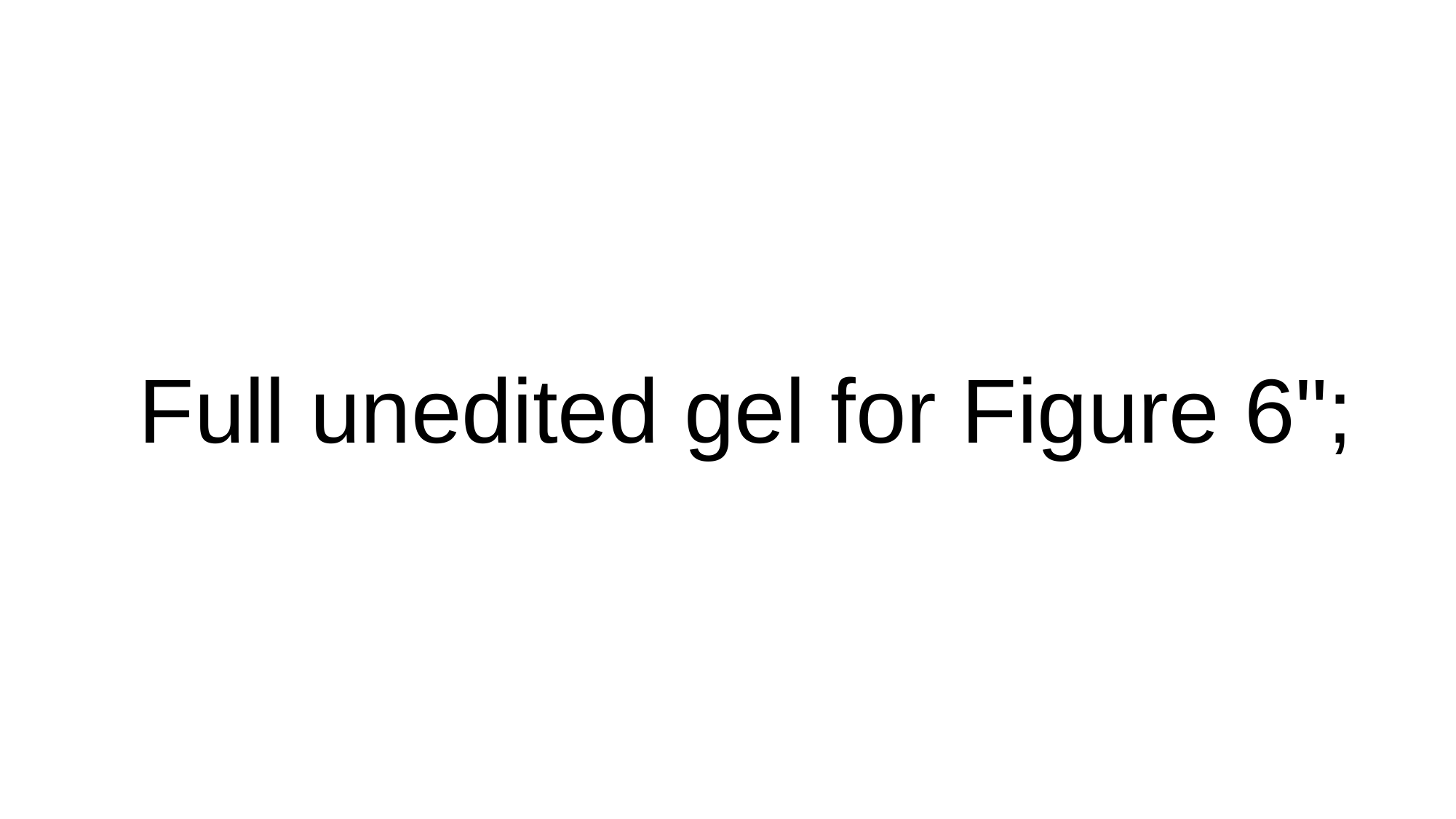

Full unedited gel for Figure 6";

## Slide 16
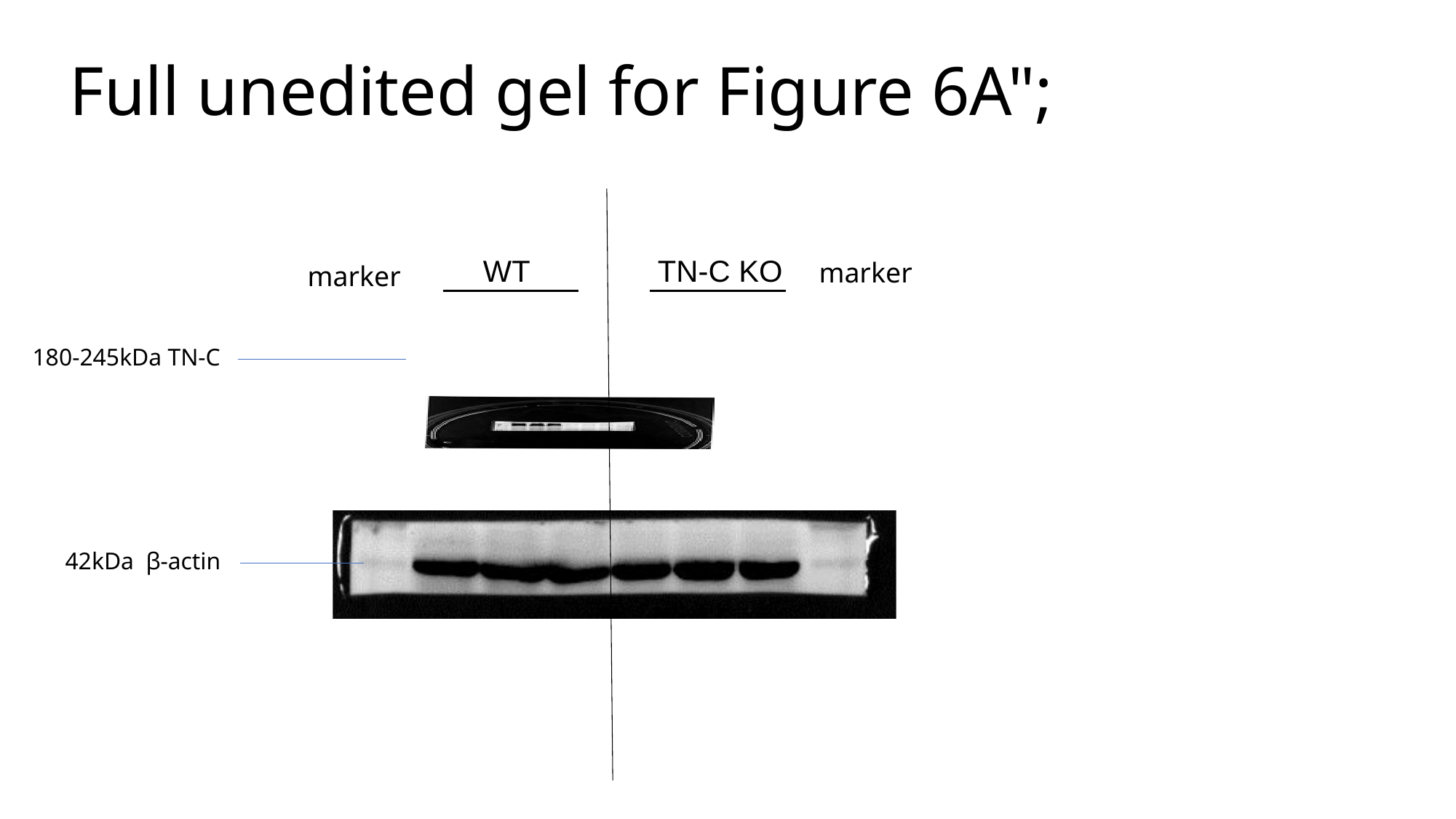

# Full unedited gel for Figure 6A";
WT
TN-C KO
marker
marker
180-245kDa TN-C
42kDa β-actin

## Slide 17
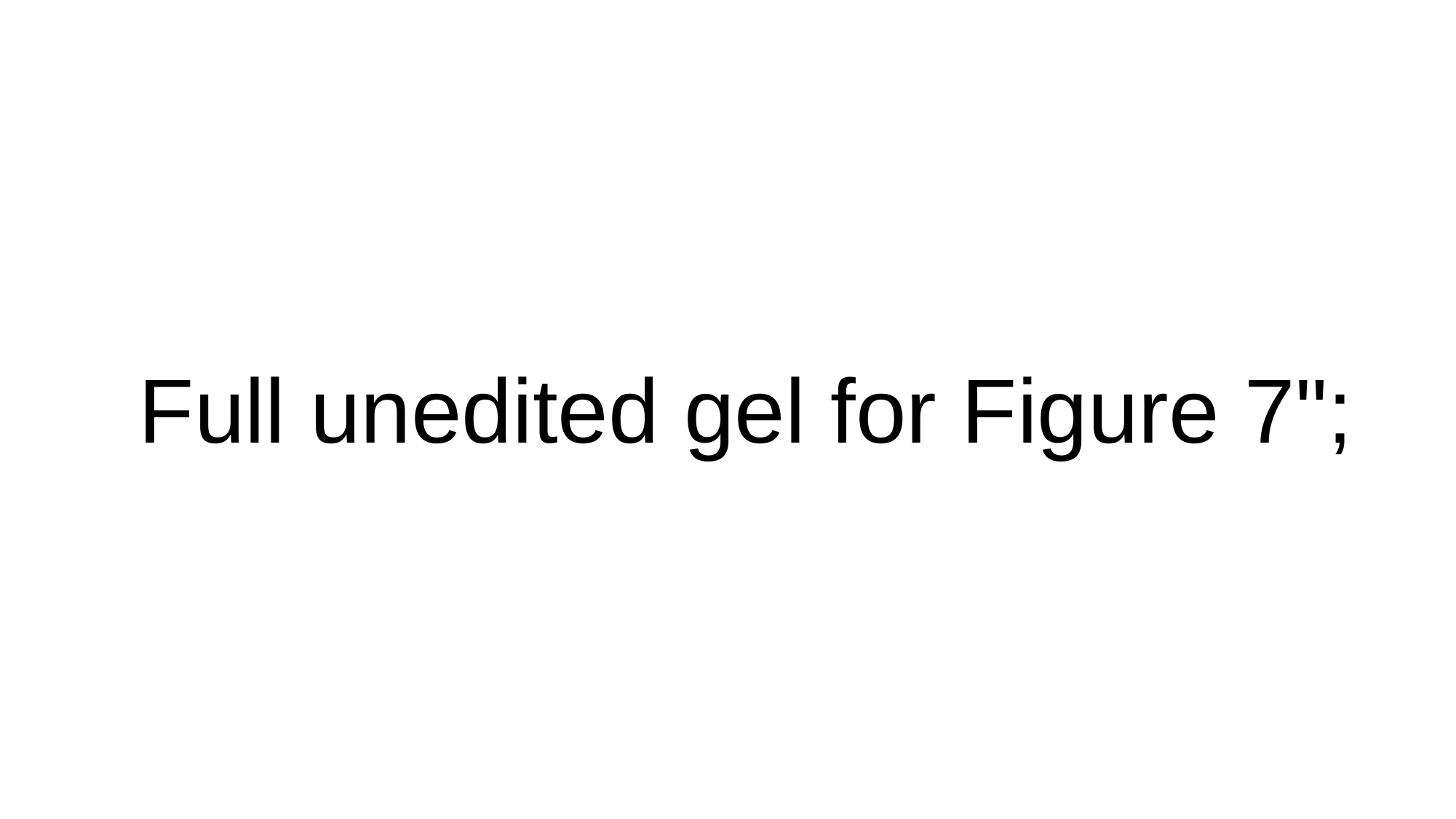

Full unedited gel for Figure 7";

## Slide 18
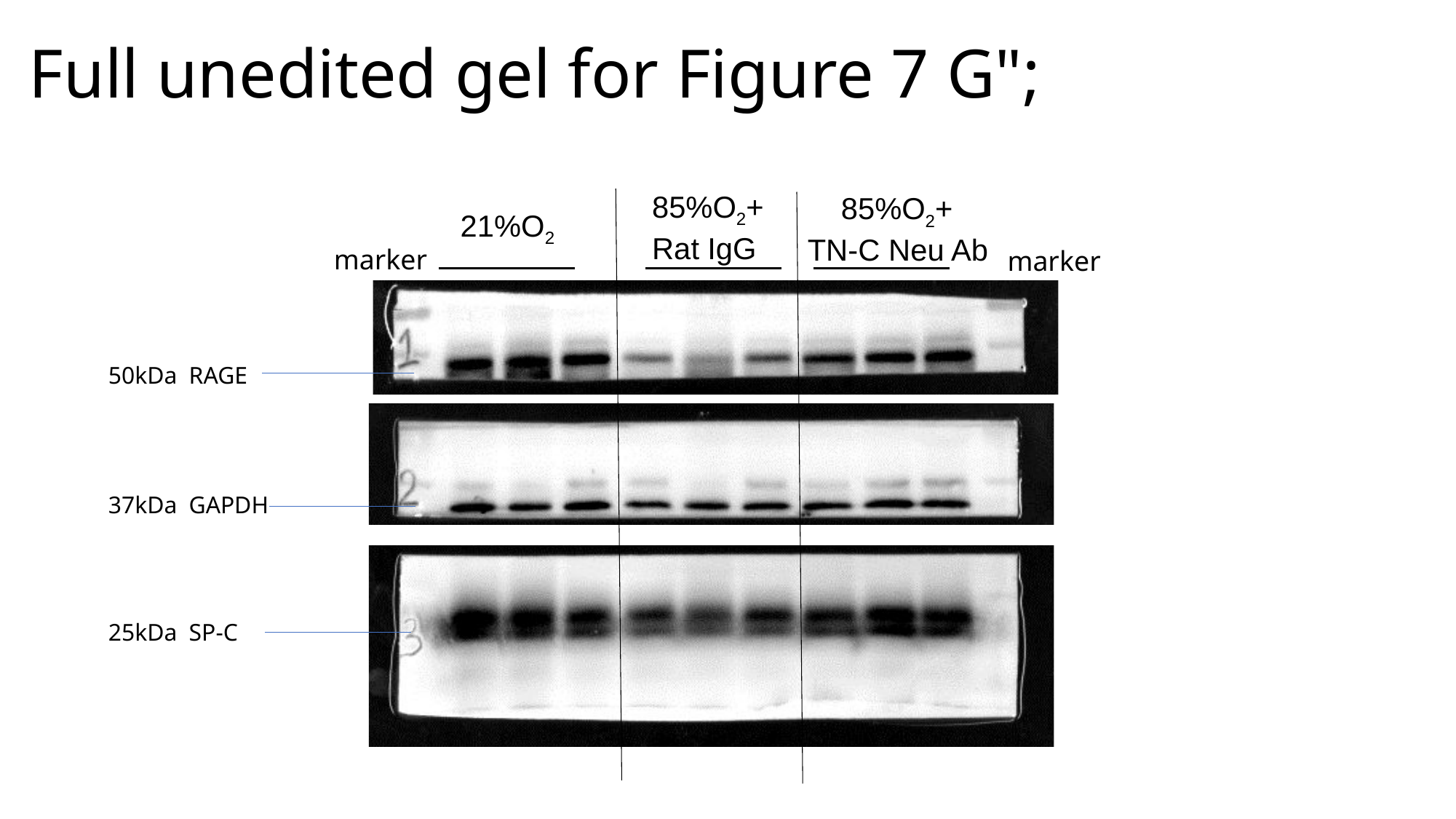

# Full unedited gel for Figure 7 G";
85%O2+
Rat IgG
 85%O2+
TN-C Neu Ab
21%O2
marker
marker
50kDa RAGE
37kDa GAPDH
25kDa SP-C
